# Thiol-Functionalized Magnetic Biochar-Mediated Matrix Cleanup and Selective Enrichment for Trace-Level Rapid Determination of Cadmium in Environmental Water and Aquatic Products

**DOI:** 10.3390/toxics14090785

**Published:** 2026-09-05

**Authors:** Lei Zhu, Xizhuang Zhu, Zhuping Liu, Longxiang Fang, Liping Qiu, Shunlong Meng, Chao Song

**Affiliations:** 1 Wuxi Fisheries College, Nanjing Agricultural University, Wuxi 214081, China; zhul@stu.njau.edu.cn (L.Z.); 15006377405@163.com (X.Z.); 2 Freshwater Fisheries Research Center, Chinese Academy of Fishery Sciences, Wuxi 214081, China; liuzhuping@ffrc.cn (Z.L.); fanglongxiang@ffrc.cn (L.F.); qiulp@ffrc.cn (L.Q.); 3 Laboratory of Quality & Safety Risk Assessment for Aquatic Products on Environmental Factors (Wuxi), Ministry of Agriculture and Rural Affairs, Wuxi 214081, China; 4 Key Laboratory of Control of Quality and Safety for Aquatic Products, Ministry of Agriculture and Rural Affairs, Beijing 100141, China

**Keywords:** cadmium (II) ion, thiol-functionalized magnetic biochar, selective enrichment, aquatic products, rapid determination

## Abstract

Efficient enrichment and rapid determination of trace cadmium (Cd^2+^) in complex environmental and food matrices remain challenging because of severe matrix interference and insufficient sample pretreatment efficiency. Herein, a thiol-functionalized silica-coated magnetic biochar (MBC@SiO_2_–SH) was developed as a selective magnetic adsorbent and integrated with a homogeneous chemiluminescence immunoassay (CLIA) to establish an enrichment-assisted analytical strategy for trace Cd^2+^ determination in environmental water and aquatic products. Owing to its abundant thiol coordination sites, large specific surface area, and excellent magnetic responsiveness, MBC@SiO_2_–SH exhibited a high practical Cd^2+^ enrichment capacity (82.65 mg/g) under high-loading conditions, remarkable selectivity, and good reusability toward Cd^2+^. Adsorption kinetic, isotherm, thermodynamic, and X-ray photoelectron spectroscopy analyses revealed that Cd^2+^ uptake was predominantly governed by Cd–S coordination accompanied by physical adsorption. Under the optimized fixed-bed enrichment conditions, the proposed column achieved a preconcentration factor of approximately 40, enabling efficient matrix cleanup and selective enrichment prior to CLIA. The integrated method exhibited excellent analytical performance for both environmental water and aquatic products, with recoveries of 92.26–118.64% and 92.41–108%, respectively, and showed good agreement with ICP-MS. By coupling selective magnetic preconcentration with rapid homogeneous immunodetection, the proposed strategy provides a simple, sensitive, and efficient analytical platform for routine monitoring of trace Cd^2+^ and offers a generalizable framework for enrichment-assisted rapid analysis of trace contaminants in complex environmental and food matrices.

## 1. Introduction

As environmental monitoring and food safety surveillance continue to move toward trace-level, rapid, and high-throughput analysis, the major bottleneck limiting analytical performance is gradually shifting from instrumental detection to sample pretreatment [1,2]. Heavy metal contamination has become a persistent environmental and food safety concern owing to its long-term persistence, bioaccumulation, and toxicity [3]. In complex environmental and food matrices, target contaminants usually exist at extremely low concentrations and coexist with abundant dissolved organic matter, proteins, lipids, and inorganic ions [4]. These matrix components not only dilute target analytes but also interfere with subsequent analytical processes, resulting in reduced sensitivity, poor reproducibility, and inaccurate quantification [2]. Therefore, developing pretreatment strategies capable of simultaneously removing matrix interference and enriching trace analytes has become a fundamental challenge for rapid and reliable contaminant analysis [5].

Among various toxic contaminants, cadmium (Cd^2+^) is of particular concern because of its high mobility, long biological half-life, and strong tendency for bioaccumulation throughout aquatic food webs [6]. Continuous exposure to cadmium has been associated with renal dysfunction, skeletal damage, and multiple chronic diseases [7]. Consequently, many countries have established increasingly stringent maximum residue limits for cadmium in aquatic products and environmental waters. Reliable determination of trace Cd^2+^ in these complex matrices is therefore essential for environmental risk assessment and food safety monitoring. However, the coexistence of complicated biological and environmental matrices greatly compromises analytical accuracy, making trace-level Cd^2+^ determination considerably more challenging than that in simple aqueous systems [8].

Currently, laboratory-based techniques such as inductively coupled plasma mass spectrometry (ICP-MS) [8], graphite furnace atomic absorption spectrometry (GFAAS) [9], and inductively coupled plasma optical emission spectrometry (ICP-OES) [10] provide excellent sensitivity and analytical accuracy for cadmium determination. Nevertheless, these methods generally require sophisticated instrumentation, labor-intensive sample preparation, and centralized laboratory facilities, limiting their applicability for rapid and high-throughput analysis. Homogeneous chemiluminescence immunoassay (CLIA), by contrast, offers rapid detection, high sensitivity, operational simplicity, and excellent compatibility with batch analysis [11]. However, its analytical performance in complex samples is frequently compromised by matrix-derived proteins, lipids, dissolved organic matter, and coexisting ions [12], which suppress chemiluminescent signals and interfere with antibody recognition. As a result, improvements in immunoassay performance are often constrained not by the detection principle itself but by insufficient sample pretreatment. Although considerable efforts have been devoted to developing more sensitive probes, antibodies, and signal amplification strategies [13,14], comparatively little attention has been paid to designing selective pretreatment materials that simultaneously eliminate matrix interference and enrich trace metal ions before rapid immunodetection.

Magnetic solid-phase extraction (MSPE) has emerged as an attractive pretreatment strategy because it enables rapid magnetic separation, efficient enrichment, and effective matrix cleanup without centrifugation or filtration [15]. Nevertheless, conventional magnetic adsorbents generally suffer from insufficient selectivity toward Cd^2+^ owing to the lack of specific binding sites. Biochar possesses a porous structure, large surface area, and excellent environmental compatibility, making it an ideal substrate for adsorbent construction [16]. However, pristine biochar mainly contains nonspecific oxygen-containing functional groups, resulting in limited affinity and selectivity for Cd^2+^. According to the Hard and Soft Acids and Bases (HSAB) theory [17], thiol groups exhibit strong coordination affinity toward soft metal ions such as Cd^2+^. Therefore, integrating magnetic separation with thiol-functionalized biochar is expected to simultaneously achieve selective Cd^2+^ enrichment and efficient matrix cleanup prior to immunoassay.

Based on this hypothesis, we developed a thiol-functionalized silica-coated magnetic biochar (MBC@SiO_2_–SH) as a selective magnetic pretreatment material and integrated it with homogeneous CLIA to establish an enrichment-assisted analytical strategy for rapid trace-level cadmium determination in environmental water and aquatic products. Unlike previously reported thiol-functionalized magnetic adsorbents that mainly focus on heavy-metal removal, MBC@SiO_2_–SH was specifically engineered for analytical enrichment by integrating magnetic separation, matrix purification, and selective Cd^2+^ capture. The hierarchical architecture consisting of Fe_3_O_4_ magnetic core, SiO_2_ protective shell, porous biochar framework, and thiol coordination sites enables efficient analyte enrichment prior to rapid immunodetection. The adsorption behavior, selectivity, adsorption mechanism, regeneration performance, and analytical enhancement capability were systematically investigated. More importantly, this work demonstrates that selective magnetic preconcentration can bridge the sensitivity gap between rapid CLIA and conventional instrumental analysis, providing a new strategy for trace contaminant monitoring in complex environmental and food matrices.

## 2. Materials and Methods

### 2.1. Chemicals and Reagents

All chemicals and solvents were of analytical grade unless otherwise specified and were used without further purification. Anhydrous ethanol, ammonia solution, and toluene were purchased from ANPEL Laboratory Technologies Inc. (Shanghai, China). Ferric chloride hexahydrate (FeCl_3_·6H_2_O) and ferrous chloride tetrahydrate (FeCl_2_·4H_2_O) were obtained from Shanghai Macklin Biochemical Co., Ltd. (Shanghai, China). Tetraethyl orthosilicate (TEOS) and 3-mercaptopropyltriethoxysilane (MPTES) were also supplied by Shanghai Macklin Biochemical Co., Ltd. (Shanghai, China). Cadmium nitrate tetrahydrate (Cd(NO_3_)_2_·4H_2_O), lead nitrate, copper acetate, zinc acetate, and chromium chloride were purchased from North Weiye Measurement Group Co., Ltd. (Xinyang, China).

Ultrapure water (18.2 MΩ·cm) was used throughout the experiments. A series of Cd^2+^ standard solutions were prepared by serial dilution of Cd(NO_3_)_2_·4H_2_O with ultrapure water. Standard solutions of competing metal ions (100 mg/L) were prepared by dissolving the corresponding metal salts in ultrapure water.

### 2.2. Preparation of BC and MBC@SiO_2_-SH

The collected moss was air-dried, further dried at 105 °C for 24 h, ground, passed through a 100-mesh sieve, and stored in a desiccator. Moss biochar (BC) was prepared via high-temperature pyrolysis: the powdered moss was tightly packed in a quartz boat and heated in a tube furnace under N_2_ atmosphere (350 mL/min) at a heating rate of 10 °C·min^−1^ to 800 °C, held for 2 h, and cooled naturally. The resulting biochar was sieved (300-mesh) and stored under dry conditions.

To prepare magnetic biochar (MBC), FeCl_3_·6H_2_O and FeCl_2_·4H_2_O (Fe^3+^: Fe^2+^ molar ratio = 2:1) were dissolved in 200 mL deionized water. Under N_2_ protection, 10 g of BC was ultrasonically dispersed into the solution in a three-necked flask. With mechanical stirring (500 rpm) at 60 °C, 40 mL of ammonia (25–28 wt%) was added dropwise to adjust the pH to 10–11, initiating coprecipitation. After 2 h of reaction, Fe_3_O_4_ particles were deposited on the biochar. The product was magnetically separated, washed with deionized water and ethanol until neutral, and vacuum-dried at 60 °C for 12 h.

For SiO_2_ coating, 10 g of MBC was dispersed in 200 mL ethanol and ultrasonicated for 30 min. Then, 100 mL deionized water and 20 mL ammonia were added as the catalyst. Under reflux at 60 °C and stirring (500 rpm), a mixture of 5 mL tetraethyl orthosilicate (TEOS) and 20 mL ethanol was added dropwise over 1.5–2 h. The reaction continued for 6 h to form a SiO_2_ shell via hydrolysis and condensation. The resulting MBC@SiO_2_ was magnetically collected, washed with ethanol–water (1:1, *v*/*v*), and vacuum-dried at 60 °C for 12 h.

Finally, thiol functionalization was conducted by drying 10 g of MBC@SiO_2_ at 105 °C for 2 h to activate surface hydroxyl groups. Under N_2_, the material was dispersed in 150 mL anhydrous toluene, followed by addition of 6 mL 3-Mercaptopropyltriethoxysilane (MPTES, ~50% excess). The reaction proceeded under reflux at 80 °C for 24 h to graft thiol groups. The product (MBC@SiO_2_-SH) was magnetically separated, washed with toluene and ethanol, and vacuum-dried at 40 °C for 24 h.

### 2.3. Characterizations of Materials

The surface microstructure of sample was analyzed using the scanning electron microscope (SEM). The Brunauer–Emmett–Teller (BET) surface area, pore size, and pore volume of the sample were analyzed with an Automatic Surface Area and Pore Analyzer. Vibrating Sample Magnetometer (VSM) primarily measures the magnetic properties of biochar to determine their magnetic classification. The surface functional group of the sample was analyzed with Fourier Transform Infrared Spectroscopy (FT-IR) spectrophotometer. The thermal stability of the materials was analyzed using a thermogravimetric analyzer (TGA). The biochar structure was characterized by X-ray Diffraction (XRD) with Cu Kα radiation, scanning was run from 5° to 90° (2θ). The surface chemical states of the catalyst were analyzed by X-ray photoelectron spectrometry (XPS) using a K-Alpha spectrometer with Al Kα radiation. The specific Characterization instruments for biochar are detailed in the Supplementary Information (SI) Text S1.

### 2.4. Cd^2+^ Adsorption by MBC and MBC@SiO_2_-SH

Subsequent adsorption experiments were performed within the environmental Cd^2+^ concentration range of 1–50 mg/L. The effects of adsorbent dosage (0.2, 0.4, 0.6, 0.8, 1, and 1.2 g/L) and solution pH (adjusted to 3, 4, 5, 6, and 7 with 0.1 mM HCl/NaOH) were studied. All samples containing the adsorbent and 30 mg/L Cd^2+^ solution were oscillated at 25 °C for 12 h to ensure adsorption equilibrium before the final Cd^2+^ concentration was measured via Inductively Coupled Plasma Mass Spectrometry (ICP-MS). Detection information for Cd^2+^ is provided in SI Text S2.

The adsorption behavior was systematically investigated through kinetic, isotherm, and thermodynamic experiments. Kinetic studies employed an adsorbent dosage of 0.18 g/L in 100 mL of 30 mg/L Cd^2+^ solution (pH 5.0, 25 °C), with samples collected from 0 to 24 h. Isotherm experiments utilized varying Cd^2+^ concentrations (5–100 mg/L) shaken for 12 h at 25 °C, and the data were fitted with Freundlich and Langmuir models. Thermodynamic studies were conducted at different temperatures (15, 25, 35, 45 °C) with an adsorbent dosage of 0.6 g/L in 30 mL of 30 mg/L Cd^2+^ solution. All samples were filtered (0.22 μm) after reaching equilibrium, and the residual Cd^2+^ concentration was quantified by ICP-MS. Detailed information about the models is provided in SI Text S3.

Also, competitive enrichment was investigated in a multicomponent system containing 30 mg/L each of Cd^2+^, Pb^2+^, Cr^3+^, Cu^2+^, and Zn^2+^, using an adsorbent dosage of 1.0 g/L under identical conditions. Additionally, adsorption–desorption cycling experiments were performed. The spent adsorbent was regenerated by shaking with 0.1 M EDTA-2NaCa·H_2_O for 2 h at 25 ± 1 °C, followed by filtration, washing with deionized water, and drying at 60 °C. The regenerated adsorbent was then reused in subsequent enrichment cycles under the initial conditions (30 mg/L Cd^2+^, pH 5.0, 25 °C, 1.0 g/L dosage).

### 2.5. Cd^2+^ Enrichment by BC and MBC@SiO_2_-SH

#### 2.5.1. Cd^2+^ Enrichment in Environmental Water

The enrichment performance was further evaluated in real environmental water matrices (lake and pond water) spiked with 5, 30, and 50 mg/L Cd^2+^. A fixed adsorbent dosage of 0.6 g/L was added to 30 mL of the water sample and shaken at 25 °C for 12 h to reach equilibrium. The residual Cd^2+^ concentration was determined by ICP-MS to calculate the enrichment capacity and removal efficiency, with all experiments conducted in triplicate. It should be noted that the Cd^2+^ uptake obtained in this enrichment experiment represents the practical Cd^2+^ loading capacity under enrichment conditions rather than the equilibrium adsorption capacity determined from batch adsorption experiments.

To investigate the applicability of the material for continuous sample pretreatment, MBC@SiO_2_–SH was packed into a solid-phase extraction (SPE) column and employed as a magnetic preconcentration medium. Briefly, 2 L of real environmental water containing 30 mg/L Cd^2+^ was continuously pumped through the column using a peristaltic pump at a constant flow rate. After adsorption, the retained Cd^2+^ was eluted with 50 mL of 0.1 M EDTA-2NaCa·H_2_O, and the eluate was subsequently subjected to CLIA analysis. The Cd^2+^ concentrations in the influent, effluent, and eluate were determined by ICP-MS.

The preconcentration factor (PF) was calculated according to Equation (1):

(1) $$PF=\frac{V_{s}\times R}{V_{e}}$$

where *V_s_* is the sample volume, *V_e_* is the elution volume, and *R* is the recovery of Cd^2+^ after the enrichment process.

#### 2.5.2. Cd^2+^ Enrichment in Aquatic Products

A 5 mL aliquot of the aquatic product sample (0.5 g), pretreated with 5% nitric acid, was transferred into a glass vial. The pH was adjusted to approximately 8.0 by adding 0.6 M NaOH, and the final volume was brought to 10 mL. Then, 40 mg of adsorbent (BC@SiO2-SH) was introduced, and the mixture was magnetically stirred for 35 min. The adsorbent was then collected and immersed in 0.1 M HCl for 10 min, followed by washing with 0.1 M EDTA-2NaCa·H_2_O and deionized water. The eluates were combined to a total volume of 20 mL, and the Cd^2+^ concentration was determined by ICP-MS.

### 2.6. Trace-Level Determination of Cadmium by CLIA

We chose a sensitive and specific CLIA method based on sandwich immunoassay. In this system, donor and acceptor microspheres are brought into proximity through antigen–antibody interactions, enabling energy transfer and light emission that can be quantitatively correlated with analyte concentration. The emitted signals are collected and processed to determine target levels.

In the experiment, 20 μL of water sample filtered through a 0.22 μm membrane was first added to an eight-well plate. Then, 20 μL of biotinylated antibody was added. Next, under light-protected conditions, 40 μL of a mixture was added, containing donor microspheres conjugated with streptavidin and acceptor microspheres conjugated with the antibody. The resulting 80 μL reaction system was incubated at 37 °C for 30 min. Finally, the sample was transferred to a detection instrument, where light signals were collected by a photosensitive element. The analyte concentration was calculated using mathematical fitting. The limit of detection (LOD) was 0.28 μg/L for Cd^2+^.

### 2.7. Data Analysis

All experiments were conducted in triplicate, and the results are expressed as mean values. Data analysis and visualization were performed using Microsoft Excel 2021, Origin Pro 2022, and MDI Jade 6.5. The normality of the data distribution was assessed using the Kolmogorov–Smirnov test, and homogeneity of variances was verified by Levene’s test.

## 3. Results and Discussion

### 3.1. Construction and Structural Characterization of MBC@SiO_2_–SH

**Figure 1:**
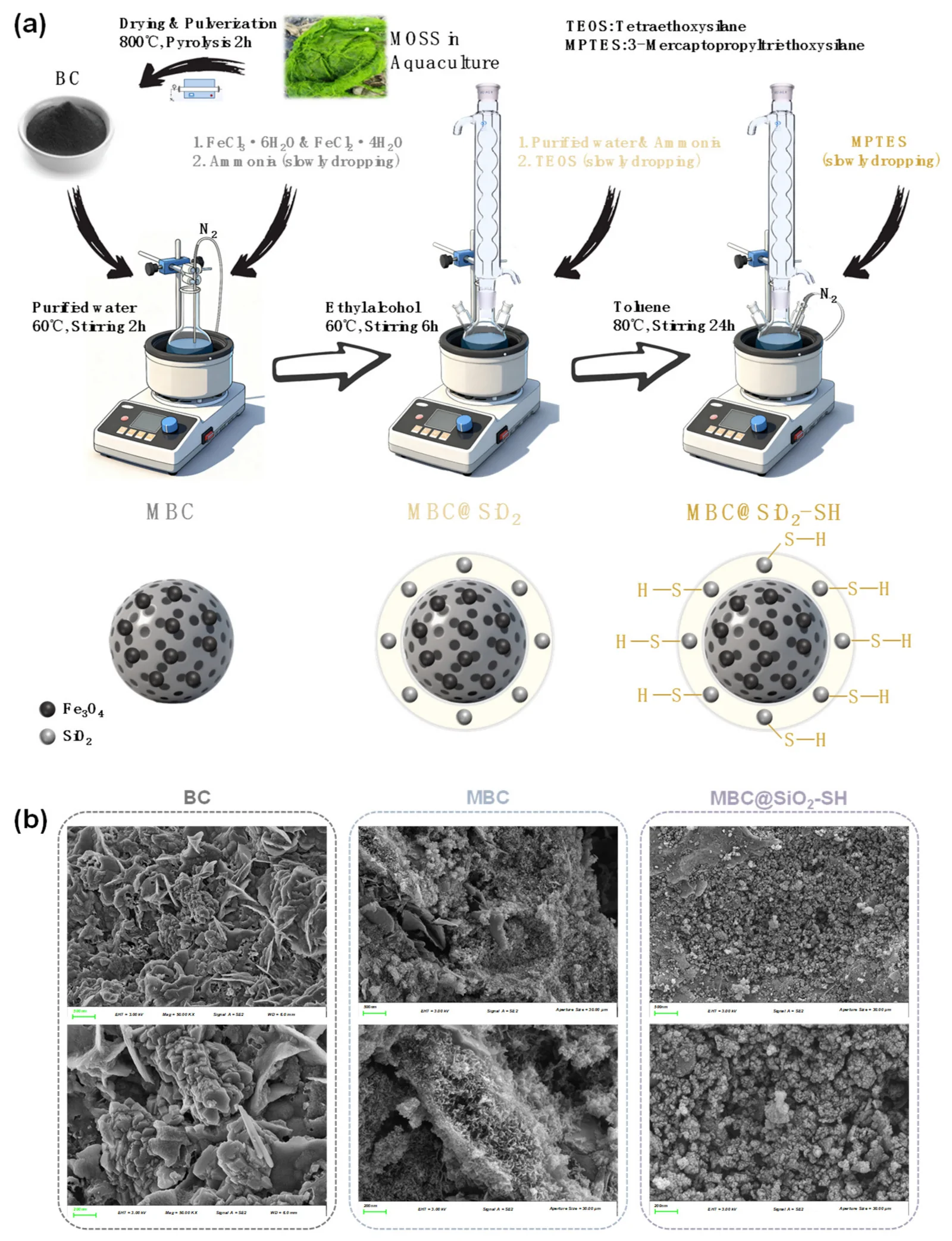
Synthesis of MBC@SiO_2_-SH. (**a**) Process diagram for preparing MBC@SiO_2_-SH from aquaculture moss raw materials. (**b**) Scanning electron microscopy (SEM) map of BC, MBC, and MBC@SiO_2_-SH.

The fabrication procedure of MBC@SiO_2_–SH is illustrated in Figure 1a. Moss-derived biochar (BC) was first magnetized through the in situ coprecipitation of Fe_3_O_4_ nanoparticles, followed by SiO_2_ coating and thiol grafting to obtain the thiol-functionalized magnetic adsorbent. This hierarchical architecture was designed to integrate magnetic responsiveness, structural stability, and abundant Cd^2+^ coordination sites into a single enrichment material. The morphological evolution is presented in Figure 1b. Pristine BC exhibited a relatively dense carbon framework with limited pore development and an uneven amorphous surface. After Fe_3_O_4_ loading, abundant magnetic nanoparticles were uniformly distributed on the carbon matrix, accompanied by the formation of a more open porous structure [18]. Subsequent SiO_2_ coating generated a continuous protective layer while preserving the interconnected pore network [19], and thiol functionalization maintained the porous architecture without structural collapse, favoring rapid Cd^2+^ diffusion and adsorption [20].

The structural optimization was further confirmed by BET analysis (Table 1). The BET surface area increased from 35.652 m^2^/g for BC to 102.413 m^2^/g for MBC@SiO_2_–SH, corresponding to an increase of approximately 187.26%. The Langmuir surface area increased from 94.735 to 756.211 m^2^/g, corresponding to approximately 698.24%, while pore volume increased from 0.044 to 0.223 cm^3^/g, corresponding to approximately 406.82%. The enlarged surface area and pore volume provide more accessible adsorption sites and facilitate mass transfer, thereby improving the enrichment efficiency toward Cd^2+^. The increase in surface area after thiol functionalization can be attributed to the combined effects of MPTES grafting and silica-supported surface reconstruction. The introduction of thiol-containing silane molecules may prevent excessive pore collapse during modification and improve the accessibility of internal porous channels, thereby exposing more adsorption domains [21]. Similar observations have been reported for thiol-modified biochar and silica-based adsorbents, where organosilane functionalization altered pore structure and increased accessible surface area, contributing to enhanced adsorption performance [22].

**Figure 2:**
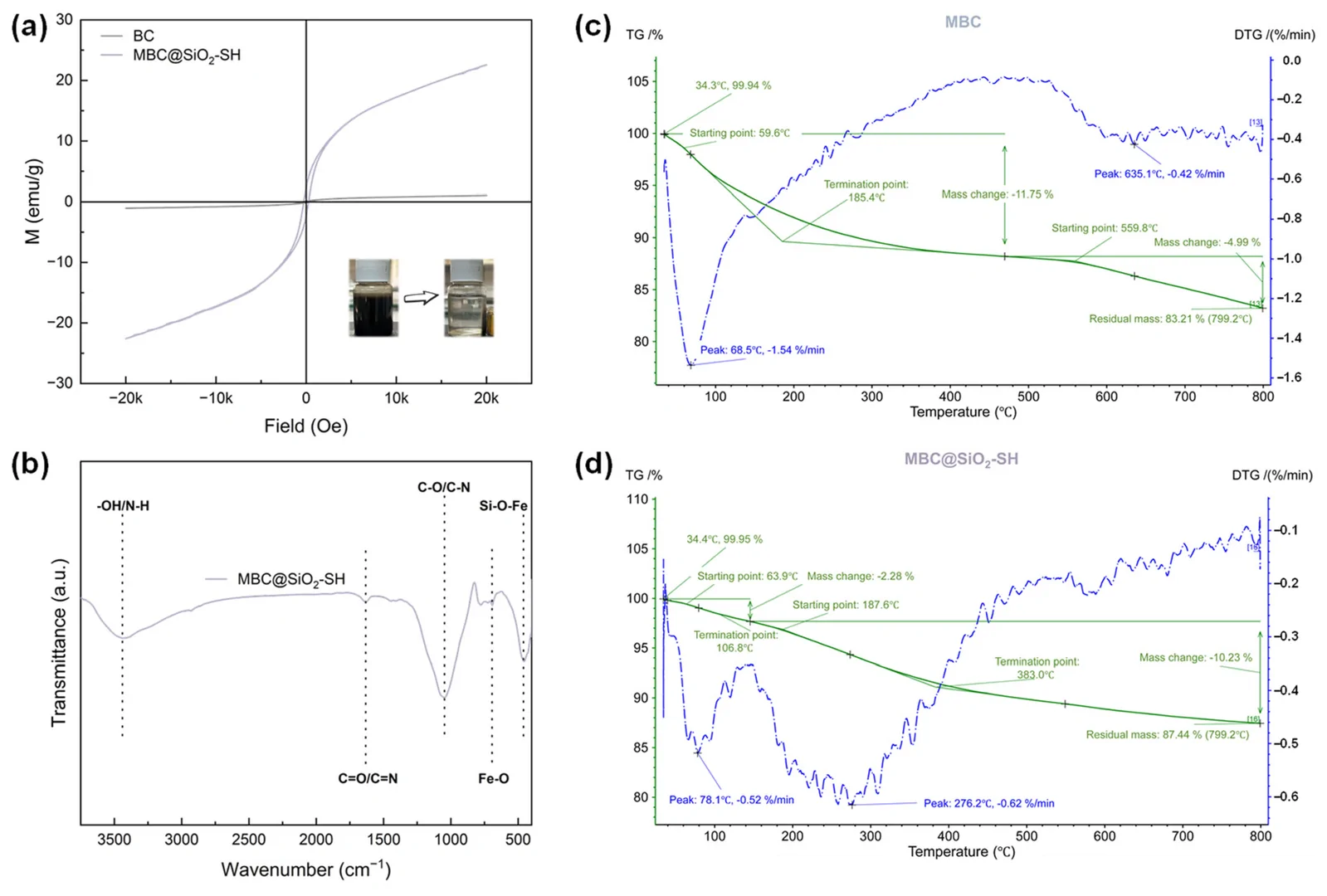
Characterization of biochar adsorbents. (**a**) The field-dependent magnetization curve of BC and MBC@SiO_2_-SH. (**b**) FT-IR spectra of MBC@SiO_2_-SH. TG/DTG curves of (**c**) MBC and (**d**) MBC@SiO_2_-SH.

Rapid magnetic separation is essential for magnetic solid-phase extraction. As shown in Figure 2a, the saturation magnetization increased from 1.05 emu/g for BC to 22.57 emu/g for MBC@SiO_2_–SH owing to the successful incorporation of Fe_3_O_4_ nanoparticles. The absence of obvious magnetic hysteresis indicates typical superparamagnetic behavior, enabling rapid magnetic recovery without residual aggregation. Successful surface functionalization was confirmed by FTIR spectroscopy (Figure 2b). The characteristic Fe–O vibration at 690.8 cm^−1^ verified the formation of magnetic iron oxide, while the Si–O–Fe vibration at 459 cm^−1^ indicated the successful deposition of the silica layer onto the Fe_3_O_4_ core. Furthermore, the presence of oxygen-containing groups together with C–S-related vibrations after MPTES modification confirmed the successful grafting of thiol groups onto the silica surface. These introduced functional groups provide abundant coordination sites for Cd^2+^ capture, particularly facilitating selective Cd–S complexation during the enrichment process. The peak assignments are in good agreement with previous reports on Fe_3_O_4_@SiO_2_-based magnetic composites. Generally, the Fe–O stretching vibration of magnetite appears in the low-wavenumber region (approximately 500–700 cm^−1^), whereas Si–O–Si and Si–O–Fe vibrations associated with silica coating are typically observed below 1100 cm^−1^ [23]. The emergence of C–S-related vibrations after MPTES functionalization further provides evidence for successful thiol immobilization on the silica shell [24].

The thermal stability of the modified material was evaluated by TGA (Figure 2c,d). Compared with MBC, MBC@SiO_2_–SH exhibited a lower total weight loss (12.51 wt% vs. 16.73 wt%) and a higher residual mass (87.44% vs. 83.21%), demonstrating that the SiO_2_ coating improved the structural stability of the magnetic composite. The DTG peak centered at 271 °C is assigned to the thermal decomposition of surface-grafted organic thiol-containing groups introduced by MPTES modification, which is consistent with the FTIR evidence of thiol functionalization. This thermal degradation profile is consistent with previous reports on silane-functionalized magnetic silica materials, where adsorbed water is typically removed at low temperatures, grafted organic moieties decompose mainly within 200–500 °C, and the inorganic SiO_2_ framework remains thermally stable [24,25].

Collectively, these results demonstrate that Fe_3_O_4_ incorporation endowed the material with excellent magnetic responsiveness, SiO_2_ coating enhanced structural robustness, and thiol grafting introduced abundant Cd^2+^ coordination sites [18,19]. Meanwhile, the significantly increased surface area and hierarchical porous framework facilitate mass transfer and adsorption, providing the structural basis for the selective enrichment and matrix-cleanup performance investigated in the following sections. Furthermore, the improved textural properties induced by thiol functionalization should be considered as an additional factor contributing to Cd^2+^ enrichment, because enlarged surface area and pore accessibility facilitate the exposure of thiol coordination sites and accelerate analyte diffusion toward active regions [21,22].

### 3.2. Structure-Dependent Adsorption Behavior

**Figure 3:**
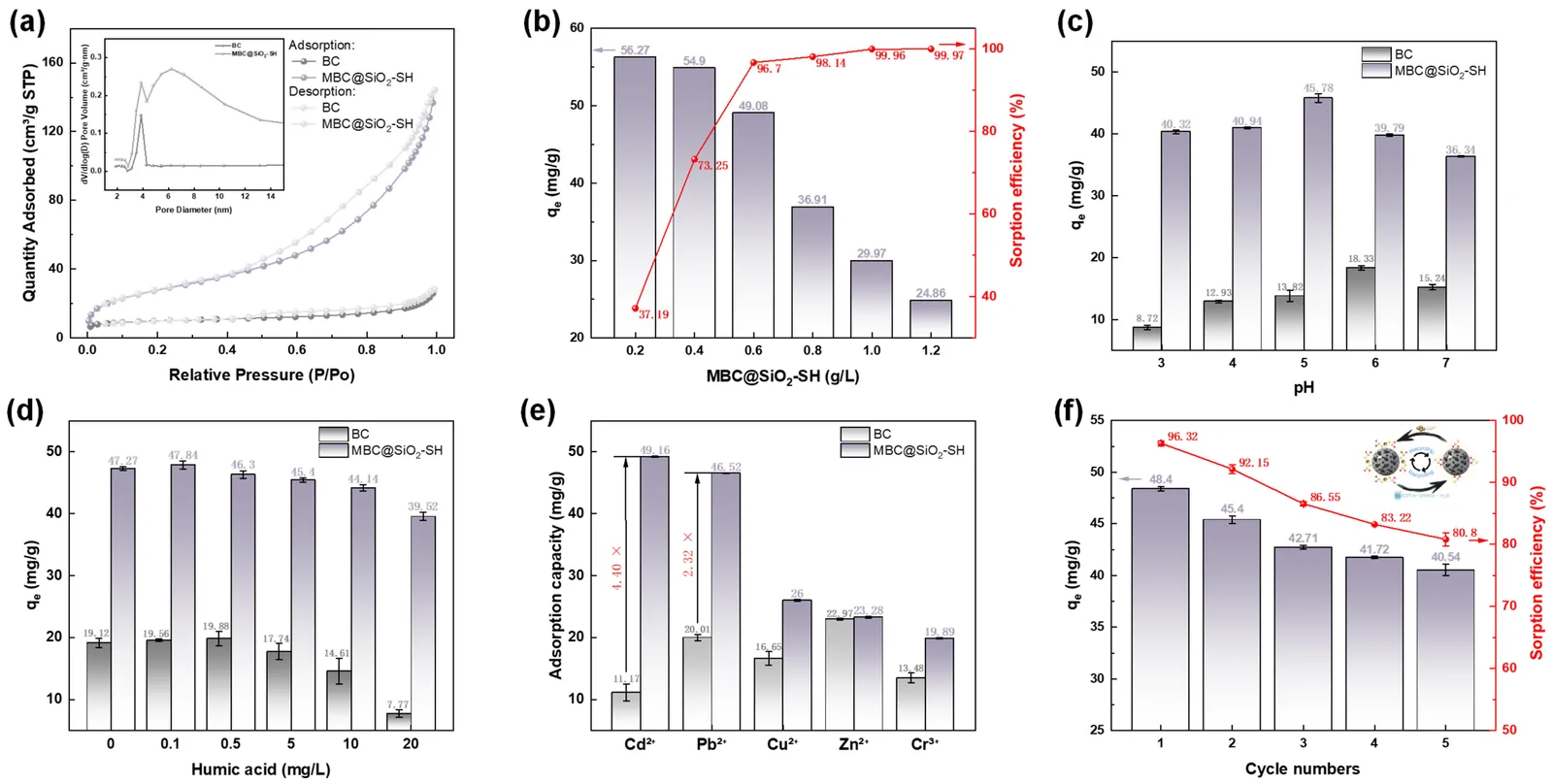
(**a**) The N_2_ adsorption–desorption isotherms (insert: the pore size distribution curves) of BC and MBC@SiO_2_-SH. Effect of (**b**) MBC@SiO_2_-SH dosage, (**c**) pH and (**d**) humic acid (HA) on Cd^2+^ adsorption by BC and MBC@SiO_2_-SH. (**e**) The saturation Cd^2+^ adsorption capacities of BC and MBC@SiO_2_-SH in the presence of an equal concentration of competitive metal ions (selective adsorption experiment). (**f**) Reusability of MBC@SiO_2_-SH.

To verify whether the structural advantages of MBC@SiO_2_–SH contributed to enhanced enrichment performance, its adsorption behavior under representative environmental conditions was systematically evaluated. As shown in Figure 3a, the N_2_ adsorption–desorption isotherm of MBC@SiO_2_–SH exhibited a typical type IV profile accompanied by an H3 hysteresis loop, indicating the presence of abundant mesoporous structures and slit-shaped pores generated from the aggregation of layered carbon domains. The gradual increase in adsorption capacity at high relative pressure regions is attributed to capillary condensation within the hierarchical mesoporous framework. This porous architecture provides accessible pathways for Cd^2+^ diffusion and facilitates adsorption by increasing the availability of surface active sites. This feature was consistent with the BET analysis, suggesting the formation of abundant accessible pores that facilitate continuous Cd^2+^ adsorption. Such a hierarchical pore architecture is favorable for Cd^2+^ enrichment, as mesopores promote the diffusion of hydrated metal ions, while micropores and external surface sites provide additional adsorption interfaces. Similar correlations between pore structure and heavy-metal adsorption performance have been widely reported for biochar-based materials, where increased surface area, well-developed pore networks, and improved accessibility of active sites contribute to enhanced metal uptake [26,27]. Therefore, the enlarged surface area and pore volume generated through Fe_3_O_4_ loading, SiO_2_ coating, and thiol functionalization likely facilitated Cd^2+^ transport and improved the utilization efficiency of adsorption sites, thereby enhancing the enrichment capability of MBC@SiO_2_–SH [28].

The adsorption performance was first optimized by varying the adsorbent dosage (Figure 3b). Increasing the dosage continuously improved the Cd^2+^ removal efficiency owing to the increased availability of adsorption sites, whereas the adsorption capacity (q_e_) gradually decreased because the initial Cd^2+^ concentration remained constant. This phenomenon is commonly observed in adsorption systems and is mainly attributed to the unsaturated adsorption sites generated at higher adsorbent concentrations, resulting in a lower adsorbate-to-adsorbent ratio [29]. Considering both removal efficiency (96.7%) and equilibrium adsorption capacity (49.08 mg/g), 0.6 g/L was selected as the optimal dosage for subsequent experiments.

Solution pH played a critical role in regulating Cd^2+^ adsorption behavior (Figure 3c). Within the investigated pH range (3–7), the adsorption capacity of MBC@SiO_2_–SH initially increased with increasing pH and reached a maximum equilibrium adsorption capacity of 45.78 mg/g at pH 5, followed by a slight decrease under more alkaline conditions. Compared with pristine BC, MBC@SiO_2_–SH maintained substantially higher adsorption capacities across the environmentally relevant pH range, demonstrating its robust Cd^2+^ enrichment capability under aquatic conditions. The enhanced adsorption at elevated pH values was mainly attributed to the progressive deprotonation of surface functional groups, particularly thiol and oxygen-containing groups, which generated more negatively charged coordination sites for Cd^2+^ binding. However, further increases in pH resulted in decreased adsorption capacity, likely due to Cd^2+^ hydrolysis and the formation of Cd hydroxyl species, which reduced the concentration of free Cd^2+^ available for surface complexation [30]. Similar pH-dependent adsorption trends have been reported for thiol-functionalized carbonaceous adsorbents, where Cd^2+^ uptake is jointly governed by surface complexation and electrostatic interactions [31].

At low pH, the protonation of surface functional groups and competition between H^+^ and Cd^2+^ for available binding sites can suppress Cd^2+^ adsorption. As the pH increases, progressive deprotonation of thiol and oxygen-containing groups increases the availability of negatively charged sites and favors Cd^2+^ surface complexation, accounting for the increase in adsorption up to pH 5. However, further increases in pH can alter the aqueous speciation of Cd^2+^ through hydrolysis, producing species such as CdOH^+^ and other hydrolyzed forms. The predominance of free Cd^2+^ at lower pH and the increasing contribution of hydrolyzed/colloidal Cd species at higher pH have been reported previously [32,33]. In addition, under sufficiently alkaline conditions, Cd(OH)_2_ precipitation may occur and contribute to the apparent removal of Cd from solution [34]. Because the present experiments were limited to pH 7, direct precipitation of bulk Cd(OH)_2_ cannot be quantitatively confirmed from the current dataset. Therefore, the slight decrease observed above pH 5 is more conservatively attributed primarily to changes in Cd^2+^ speciation and the resulting decrease in freely available Cd^2+^ for specific surface complexation, with hydroxide formation/precipitation considered a possible secondary contribution. This interpretation is consistent with previous studies showing that Cd^2+^ adsorption on biochar is strongly pH-dependent and is controlled by the combined effects of surface deprotonation, surface complexation, ion exchange, and Cd hydroxide-related processes [35].

Because dissolved organic matter is ubiquitous in natural waters, the influence of humic acid (HA) was further investigated (Figure 3d). For BC, low HA concentrations (0.1–0.5 mg/L) slightly increased the adsorption capacity from 19.12 to 19.88 mg/g, whereas higher HA concentrations (5–20 mg/L) reduced the adsorption capacity by 59.36%, probably because excessive HA molecules blocked pores and occupied active adsorption sites [36]. The inhibitory effect of high HA concentrations has also been observed in other biochar adsorption systems, where macromolecular organic matter competes with metal ions for adsorption sites or forms surface coatings that hinder ion diffusion into internal pores [37]. In contrast, the relatively small decrease observed for MBC@SiO_2_–SH at high HA concentrations indicates improved resistance toward organic interference. This behavior may be attributed to the specific coordination affinity between thiol groups and Cd^2+^, which provides stronger binding interactions than nonspecific adsorption involving oxygen-containing groups. Previous studies have demonstrated that sulfur-functionalized adsorbents maintain high Cd^2+^ selectivity even in complex matrices containing competing organic matter and coexisting ions [38].

Selectivity is essential for practical enrichment in complex matrices [39]. As shown in Figure 3e, MBC@SiO_2_–SH exhibited the highest adsorption toward Cd^2+^, followed by Pb^2+^, whereas the adsorption of Cu^2+^, Zn^2+^, and Cr^3+^ was considerably lower. The selectivity coefficients toward Cd^2+^ relative to Pb^2+^, Cu^2+^, Zn^2+^, and Cr^3+^ were 1.06, 1.89, 2.11, and 2.47, respectively. Compared with BC, the adsorption enhancement factors of MBC@SiO_2_–SH reached 4.40 for Cd^2+^, 2.32 for Pb^2+^, 1.56 for Cu^2+^, and 1.48 for Cr^3+^, confirming that thiol functionalization substantially strengthened both adsorption affinity and Cd^2+^ selectivity [40]. The regeneration performance of the adsorbent was further evaluated (Figure 3f). Adsorbed Cd^2+^ was desorbed using 0.1 mol/L EDTA-2Na containing Ca^2+^, where the stronger Cd–EDTA complex (log K ≈ 16.5) displaced Cd^2+^ from the thiol coordination sites (log K ≈ 10^–12^) [41]. After five consecutive adsorption–desorption cycles, the adsorption capacity of MBC@SiO_2_–SH decreased from 48.40 mg/g (96.32% removal) to 40.54 mg/g (80.80% removal), corresponding to an adsorption capacity retention of 83.76%. Previous studies have demonstrated that thiol-functionalized carbonaceous materials remove Cd^2+^ mainly through strong surface complexation and Cd–S coordination, which provides high adsorption affinity but may also limit the complete regeneration of some active sites due to the stability of metal–sulfur interactions [31,42]. In addition, sulfur functionalization and repeated regeneration processes may influence pore accessibility and surface characteristics, contributing to gradual capacity attenuation [42]. Nevertheless, MBC@SiO_2_–SH maintained high adsorption efficiency after repeated use, demonstrating satisfactory regeneration stability and practical applicability [43,44].

Overall, the adsorption results demonstrate that the structural advantages introduced by Fe_3_O_4_ loading, SiO_2_ coating, and thiol functionalization not only enhance adsorption capacity but also improve anti-interference ability, Cd^2+^ selectivity, and regeneration stability [45]. These characteristics collectively provide the performance basis for the selective enrichment of trace Cd^2+^ in complex environmental and food matrices.

### 3.3. Mechanistic Insight into Selective Cd^2+^ Enrichment

#### 3.3.1. Adsorption Kinetics

**Figure 4:**
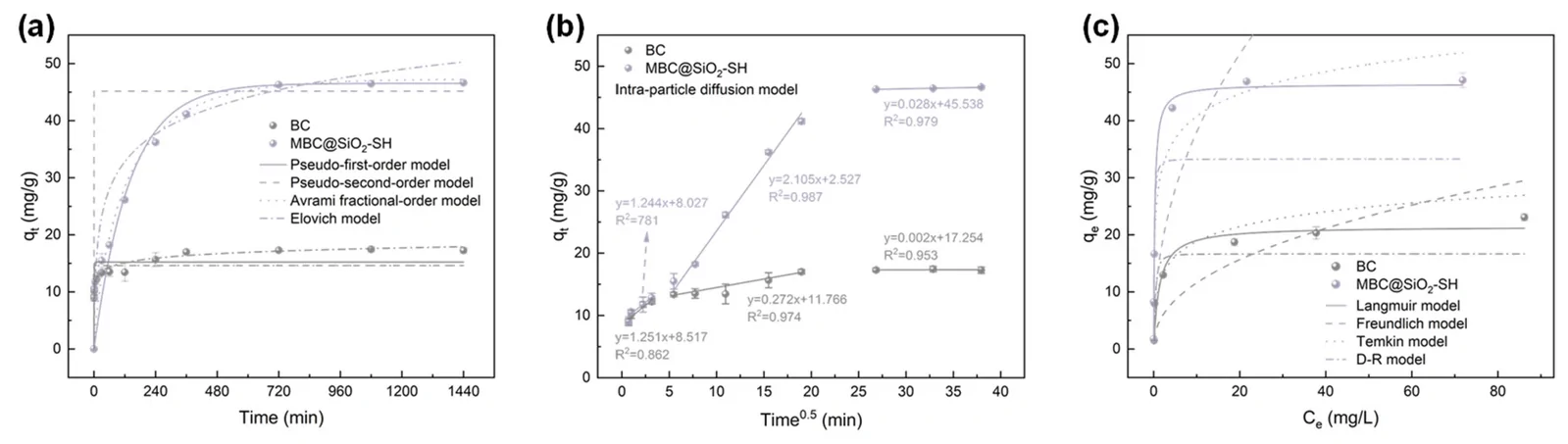
(**a**,**b**) Cd^2+^ adsorption kinetics and (**c**) the equilibrium adsorption isotherms of BC and MBC@SiO_2_-SH in aqueous solution at pH 5.0.

To elucidate the rate-limiting steps governing Cd^2+^ enrichment, the adsorption kinetics of MBC@SiO_2_–SH were systematically investigated. The experimental data were fitted using pseudo-first-order, pseudo-second-order, Avrami fractional-order, Elovich, and intra-particle diffusion models (Figure 4a,b and Table 2) [46].

As shown in Figure 4a, Cd^2+^ adsorption proceeded rapidly during the initial stage and gradually approached equilibrium after approximately 480 min, reaching an adsorption capacity of 45.04 mg/g, while complete equilibrium was achieved at 720 min. The rapid initial uptake is attributed to the abundant accessible adsorption sites distributed on the external surface and within the interconnected pore channels of MBC@SiO_2_–SH [47]. As adsorption proceeded, the remaining Cd^2+^ ions gradually diffused into the internal porous structure, where the strong coordination interaction between Cd^2+^ and thiol groups became the dominant rate-controlling process, thereby extending the equilibrium time [48].

The kinetic parameters summarized in Table 2 indicate that both the pseudo-first-order and Avrami fractional-order models provided excellent fits to the experimental data, with equilibrium adsorption capacities of 46.65 and 47.32 mg/g, respectively, and identical correlation coefficients (R^2^ = 0.999). The excellent agreement between the experimental and calculated adsorption capacities confirms the high reliability of these models in describing the adsorption process. The Avrami model provided a better description of the adsorption kinetics, suggesting that Cd^2+^ adsorption involved multiple rate-controlling steps, including diffusion processes and interactions with surface active sites [46].

The intra-particle diffusion analysis further revealed that thiol functionalization significantly increased the diffusion rate constants (k_s2_ and k_s3_) compared with pristine BC. This improvement is closely associated with the enlarged specific surface area, increased mesoporous volume, and interconnected pore structure established during Fe_3_O_4_ loading and SiO_2_ coating. These structural characteristics effectively shorten the diffusion pathway of Cd^2+^ and increase the accessibility of internal active sites, thereby facilitating rapid mass transfer throughout the adsorption process [49].

The enhanced adsorption kinetics are further supported by the surface chemistry characterized in Section 3.1. The abundant oxygen-containing groups together with the grafted thiol groups provide a high density of coordination sites, enabling rapid capture of Cd^2+^ during the initial adsorption stage while maintaining continuous adsorption within the internal porous framework. These results indicate that the hierarchical pore structure primarily accelerates Cd^2+^ transport, whereas thiol coordination governs the subsequent selective binding process.

#### 3.3.2. Adsorption Isotherms

Adsorption isotherm analysis was conducted to further elucidate the interaction between Cd^2+^ and the functionalized adsorbent at equilibrium. Four classical isotherm models, including the Langmuir, Freundlich, Temkin, and Dubinin–Radushkevich (D–R) models, were employed to fit the experimental data (Figure 4c and Table 3) [29].

Among the four models, the Langmuir model provided the best description of Cd^2+^ adsorption on MBC@SiO_2_–SH, with a correlation coefficient of R^2^ = 0.997, substantially higher than that obtained using the Freundlich model (R^2^ = 0.584). This result indicates that Cd^2+^ adsorption predominantly occurs through monolayer coverage on energetically homogeneous active sites rather than through heterogeneous multilayer adsorption [50]. Considering the successful grafting of abundant thiol groups demonstrated by FTIR, the Langmuir behavior strongly suggests that these uniformly distributed thiol coordination sites dominate the equilibrium adsorption process [51].

The Langmuir fitting further yielded a maximum equilibrium adsorption capacity (q_max_) of 46.41 mg/g, which is more than twice that of pristine BC (21.46 mg/g), demonstrating that thiol functionalization substantially enhances the utilization efficiency of adsorption sites. This remarkable increase agrees well with the enlarged specific surface area and pore volume discussed in Section 3.1, indicating that both structural optimization and surface functionalization contribute synergistically to Cd^2+^ enrichment [26,27].

The Temkin model further revealed that the equilibrium binding constant (K_t_) of MBC@SiO_2_–SH reached 136.055 L/g, which is 9.09 times higher than that of BC. This substantial increase reflects a much stronger interaction between Cd^2+^ and the modified adsorbent after thiol functionalization. Unlike pristine BC, where Cd^2+^ adsorption mainly relies on electrostatic attraction and weak complexation with oxygen-containing functional groups, the introduced thiol groups provide high-affinity coordination sites capable of forming stable Cd–S coordination bonds [52]. Consequently, MBC@SiO_2_–SH exhibits significantly greater adsorption driving force and stronger affinity toward Cd^2+^, particularly under low-concentration conditions relevant to trace enrichment.

Furthermore, the D–R model was further applied to evaluate the adsorption energy and adsorption mechanism. The mean adsorption energy (E) was calculated using E = (2β)^−1/2^, where β represents the D–R constant (mol^2^ kJ^−2^). The calculated E values were 6.72 and 11.55 kJ mol^−1^ for BC and MBC@SiO_2_–S, respectively. The higher E value of MBC@SiO_2_–S suggests stronger interactions between Cd^2+^ and the modified surface. Combined with FTIR and XPS results, the enhanced Cd^2+^ adsorption was mainly attributed to surface complexation and coordination interactions involving thiol groups, accompanied by electrostatic attraction and pore filling effects. The improved energetic uniformity further facilitates selective Cd^2+^ adsorption by minimizing the heterogeneity of adsorption sites.

Overall, the equilibrium adsorption results demonstrate that the exceptional Cd^2+^ enrichment capability of MBC@SiO_2_–SH originates from the synergistic effect of structural optimization and surface functionalization. The hierarchical porous framework improves mass transport and site accessibility, whereas uniformly distributed thiol groups dominate equilibrium adsorption through strong Cd–S coordination. Unlike conventional magnetic biochar adsorbents that mainly rely on nonspecific adsorption by oxygen-containing groups (Table S1), MBC@SiO_2_–SH provides selective chemical recognition toward Cd^2+^, which is essential for subsequent analytical enrichment in complex matrices [50,52].

#### 3.3.3. Adsorption Thermodynamics

Thermodynamic parameters, including enthalpy change (ΔH^0^), Gibbs free energy change (ΔG^0^), and entropy change (ΔS^0^), were calculated to evaluate the energetics and spontaneity of Cd^2+^ adsorption on the biochar materials. As shown in Figure S1 and Table 4, the positive values of ΔH^0^ indicate an endothermic adsorption process, which is consistent with the isotherm results. The negative ΔG^0^ values confirm the spontaneity of adsorption, while the positive ΔS^0^ values suggest an increase in randomness at the solid–liquid interface during the process. The adsorption capacity increased with rising temperature (15–45 °C), a trend also observed in other systems, such as Cd^2+^ adsorption on granular activated carbon [53]. For the thermodynamic analysis, the equilibrium constant was defined from the Langmuir equilibrium relationship at each temperature and converted to a dimensionless standard equilibrium constant before being used in the thermodynamic equations. The Langmuir constant was first expressed using molar units, and the standard concentration was defined as 1 mol L^−1^. For the dilute Cd^2+^ solutions used here, the activity coefficient was approximated as unity. The resulting standard equilibrium constant was then normalized using the defined standard state to obtain a dimensionless value before calculation of the thermodynamic parameters. The Gibbs free energy change was calculated using the dimensionless standard equilibrium constant, while the enthalpy and entropy changes were obtained from the corresponding linear van’t Hoff relationship. This explicit standard-state treatment avoids directly substituting a dimensional concentration-based equilibrium constant into the logarithmic thermodynamic equations [54,55].The increase in adsorption capacity with temperature and the positive ΔH^0^ indicate the endothermic nature of Cd^2+^ uptake. However, the positive ΔH^0^ value alone does not constitute definitive evidence of chemisorption. Instead, the thermodynamic results should be interpreted together with the FTIR, XPS, isotherm, and kinetic evidence. Collectively, these results support an important contribution from specific surface interactions and Cd–S coordination, while other interactions, including electrostatic attraction and pore filling, may also contribute to the overall adsorption process [56].

The ΔS^0^ and ΔH^0^ values for MBC@SiO_2_–SH were determined to be 171.60 J/mol*K and 27.91 kJ/mol, which are 2.33 and 1.73 times those of BC, respectively. The thermodynamic parameters indicate that Cd^2+^ adsorption on MBC@SiO_2_–SH is endothermic with a favorable positive entropy change. While these parameters alone cannot definitively establish the adsorption mechanism, their larger magnitudes—when considered alongside spectroscopic and adsorption data—are consistent with strong Cd–S coordination imparted by thiol functionalization, though concurrent physisorption and pore-filling effects cannot be excluded. Thus, the thermodynamic evidence supports, rather than independently proves, the role of chemical coordination in Cd^2+^ uptake [55,56]. This is further corroborated by the contrasting behavior of BC, which exhibits enthalpy-driven physisorption (exothermic but entropically unfavorable) [57], underscoring the specific chemisorption conferred by thiol groups.

#### 3.3.4. Structural Evidence for the Adsorption Mechanism

**Figure 5:**
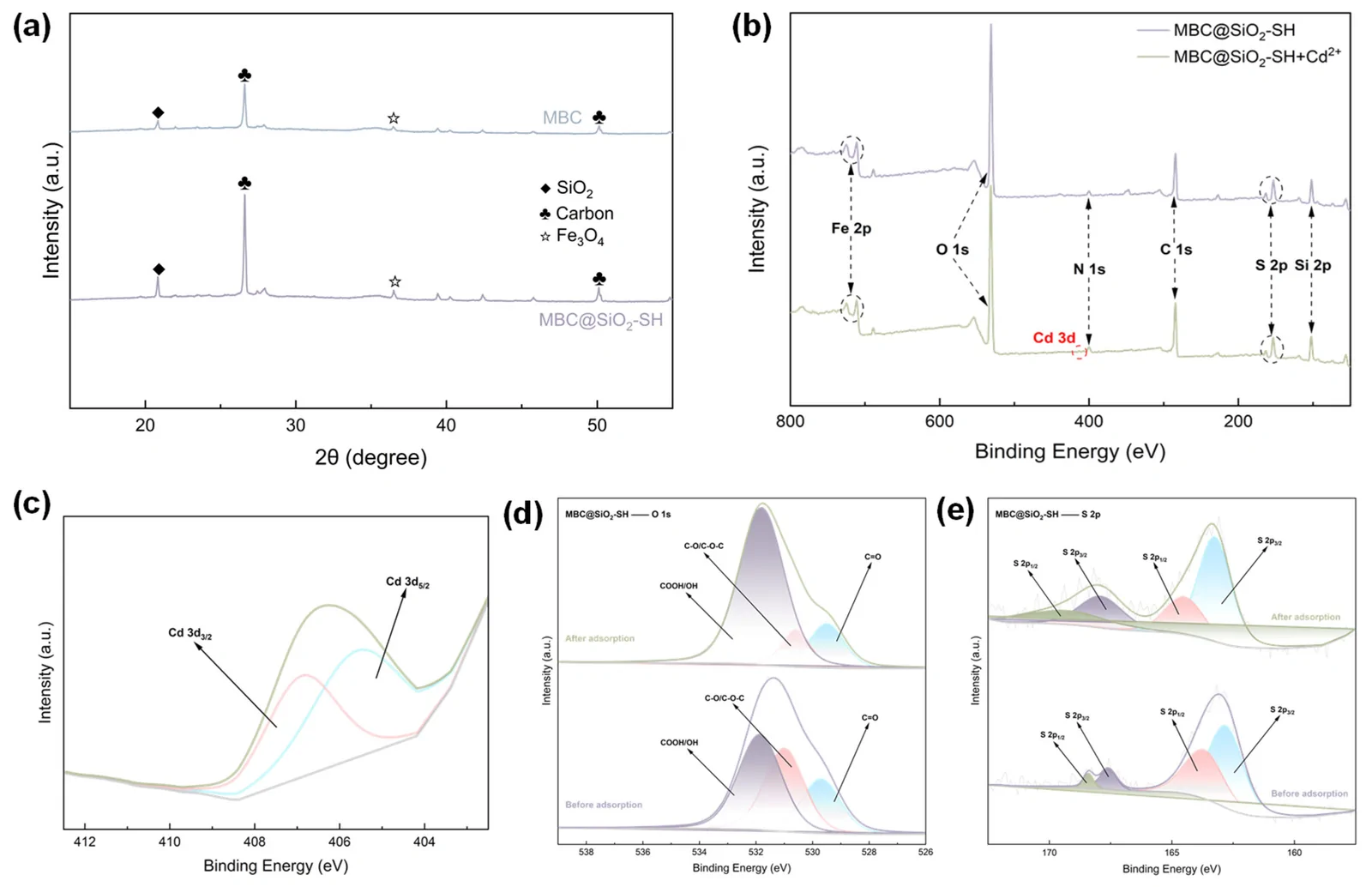
(**a**) XRD patterns of MBC and MBC@SiO_2_-SH. (**b**) XPS spectra of MBC@SiO_2_-SH before and after adsorption of Cd^2+^. (**c**) Cd 3d, (**d**) O 1s, and (**e**) S 2p.

The crystal structure evolution of the adsorbents was investigated by XRD analysis (Figure 5a). Compared with pristine BC, Fe-loaded MBC exhibited distinct diffraction peaks corresponding to Fe_3_O_4_, confirming the successful formation of magnetic iron oxide on the biochar surface. This phase transformation is consistent with the reported magnetization pathway involving the sequential conversion of FeCl_3_ to Fe(OH)_3_, FeO(OH), and finally Fe_3_O_4_ [23]. Meanwhile, the weakened intensity of the amorphous carbon peak in MBC suggested strong interactions between iron species and the carbon matrix, which may contribute to the generation of additional adsorption sites and provide magnetic responsiveness for efficient separation. After SiO_2_ coating and thiol functionalization, MBC@SiO_2_–SH exhibited a more pronounced amorphous background, which was mainly attributed to the overlapping diffraction contributions from the silica shell and carbon matrix. The characteristic Fe_3_O_4_ reflections remained unchanged, indicating that the silica coating effectively preserved the crystalline integrity of the magnetic core by preventing oxidation and structural degradation. The diffraction peak assignments were consistent with standard crystallographic data and previous studies on Fe_3_O_4_-based magnetic composites, where Fe_3_O_4_ reflections correspond to the inverse spinel structure and the absence of distinct SiO_2_ peaks is associated with the amorphous nature of silica [58]. These results confirm the successful construction of a stable SiO_2_-coated magnetic biochar composite, which is consistent with the enhanced magnetic stability observed from the hysteresis loop analysis.

XPS analysis was further performed to obtain direct surface-chemical evidence for Cd^2+^ uptake and to compare MBC@SiO_2_–SH before and after adsorption (Figure 5b). After Cd^2+^ adsorption, two characteristic Cd 3d peaks appeared at 404.78 and 411.58 eV, assigned to Cd^2+^ 3d_5_/_2_ and 3d_3_/_2_, respectively (Figure 5c), directly confirming the presence of Cd on the adsorbent surface. The O 1s peak shifted slightly from 529.7 to 529.5 eV after adsorption (Figure 5d), suggesting a change in the local electronic environment of oxygen-containing surface species. This observation is consistent with possible involvement of O-containing groups in Cd^2+^ interactions, although the small shift does not by itself establish a specific Cd–O bonding configuration. Similar surface-chemical changes have been used as supporting evidence for metal-ion complexation on functionalized carbonaceous adsorbents [31,33].

The S 2p spectrum provides stronger evidence for the role of the grafted thiol groups. The sulfur signal characteristic of the thiol-containing surface decreased from 54.44% to 46.82% after Cd^2+^ adsorption (Figure 5e). Although changes in normalized XPS atomic percentages can also be affected by surface composition and sampling depth, the decrease in the sulfur-related signal after Cd^2+^ uptake is consistent with participation of the S-containing groups in Cd^2+^ binding. This interpretation agrees with previous studies showing that thiol-functionalized carbonaceous and silica-based adsorbents exhibit enhanced Cd^2+^ affinity through sulfur-containing coordination sites [31,40,42]. The newly appeared N 1s signal at 405.6 eV is attributed to nitrate species originating from Cd(NO_3_)_2_·4H_2_O and is not considered evidence for the adsorption mechanism.

Taken together, the before and after XPS results provide direct evidence for Cd^2+^ immobilization at the MBC@SiO_2_–SH surface and support the involvement of sulfur- and, to a lesser extent, oxygen-containing surface groups. The FTIR spectrum available in this study was collected for the pristine MBC@SiO_2_–SH material and was used to verify the successful introduction of thiol-containing functionalities; because a post-adsorption FTIR spectrum was not obtained, FTIR cannot be used here to claim adsorption-induced peak shifts. This limitation is now explicitly recognized. Nevertheless, the combination of thiol functionalization evidence, XPS changes after Cd^2+^ adsorption, the higher Cd^2+^ affinity of MBC@SiO_2_–SH than BC, and the pH-dependent adsorption behavior provides convergent support for specific surface interactions, particularly Cd–S coordination, as an important contributor to Cd^2+^ uptake, in agreement with previous reports on thiol-modified biochar and thiol-functionalized adsorbents [40,42].

**Figure 6:**
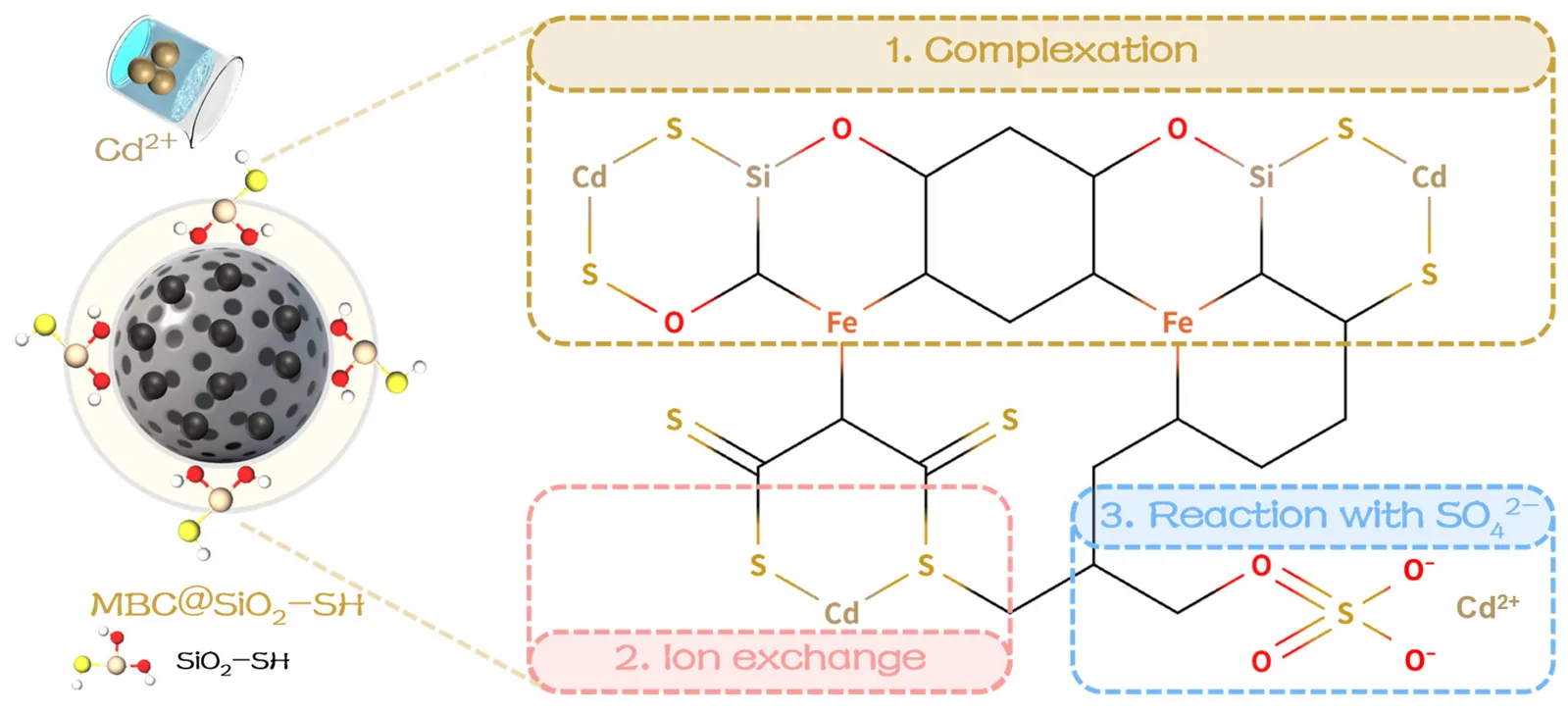
Conceptual schematic illustration of the proposed architecture of MBC@SiO_2_-SH and the plausible adsorption mechanism of Cd^2+^.

Accordingly, the adsorption mechanism proposed in Figure 6 is presented as a plausible multi-step process rather than as a uniquely demonstrated pathway. The hierarchical porous structure and the increased surface area/pore volume are expected to facilitate Cd^2+^ transport and access to adsorption sites, which is consistent with the intraparticle-diffusion analysis and the rapid initial uptake [33,47]. Electrostatic attraction may also contribute because adsorption increased with pH as surface functional groups became progressively less protonated; however, no direct surface-charge measurement was performed in the present study, so its contribution cannot be quantitatively separated. Physical adsorption/pore filling may coexist with specific surface interactions, as suggested by the D–R energy range and the heterogeneous porous structure, but these processes cannot be independently quantified from the current dataset. In contrast, the appearance of Cd 3d signals together with the post-adsorption changes in O 1s and S 2p provides the strongest experimental support for specific surface binding, with thiol-mediated Cd–S interaction likely playing an important role [31,40,42]. Therefore, the overall Cd^2+^ uptake is more appropriately described as a combination of pore-assisted mass transfer and surface interactions, with Cd–S coordination being the best-supported specific binding contribution.

### 3.4. Selective Enrichment in Complex Matrices

Although adsorption experiments demonstrated the intrinsic affinity of MBC@SiO_2_–SH toward Cd^2+^, practical rapid analysis requires efficient matrix cleanup and analyte preconcentration prior to immunodetection. Therefore, the enrichment performance of MBC@SiO_2_–SH was further evaluated in real environmental matrices to investigate its capability for selectively capturing and concentrating Cd^2+^ from complex samples.

#### 3.4.1. Selective Enrichment of Cd^2+^ in Real Environmental Water Samples

Environmental water samples, including lake water and aquaculture water (Figure S2), were selected as representative complex matrices because they contain abundant dissolved organic matter, nutrients, and competing ions that may interfere with Cd^2+^ detection. To evaluate the enrichment capability under realistic conditions, samples were spiked with Cd^2+^ at concentrations of 5, 30, and 50 mg/L and treated with MBC@SiO_2_–SH under optimized adsorption conditions. After magnetic separation, the residual Cd^2+^ concentration was determined by ICP-MS, and the enrichment capacity was calculated.

**Figure 7:**
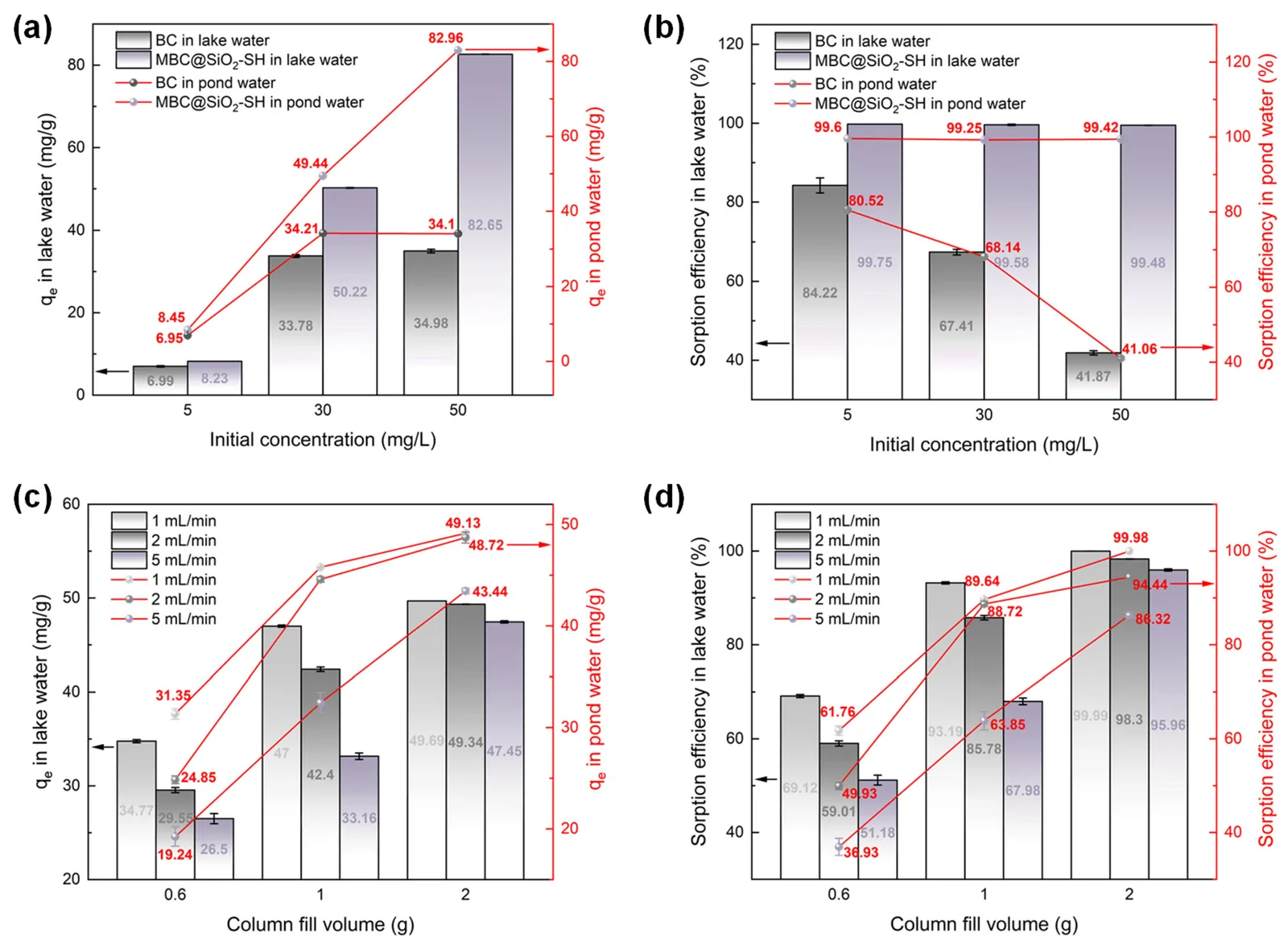
Batch and Column Adsorption Performance of MBC@SiO_2_-SH in Real Water Samples. (**a**) Adsorption capacities of BC and MBC@SiO_2_-SH in lake water and pond water. (**b**) Corresponding Cd^2+^ removal efficiencies. (**c**) Column adsorption capacities of MBC@SiO_2_-SH at different packing amounts and flow rates. (**d**) Corresponding Cd^2+^ removal efficiencies during column adsorption.

As shown in Figure 7a,b, MBC@SiO_2_–SH exhibited efficient Cd^2+^ enrichment in both lake water and aquaculture water matrices. Compared with pristine BC, MBC@SiO_2_–SH maintained significantly enhanced enrichment performance, particularly at higher Cd^2+^ concentrations. For example, in lake water containing 30 mg/L Cd^2+^, the enrichment capacities of BC and MBC@SiO_2_–SH were 33.78 and 50.22 mg/g, respectively. When the Cd^2+^ concentration increased to 50 mg/L, MBC@SiO_2_–SH achieved a practical Cd^2+^ loading of 82.65 mg/g during the enrichment process, whereas BC exhibited limited enhancement due to the saturation of nonspecific adsorption sites. This value represents the Cd^2+^ capture capability under high-loading enrichment conditions rather than the equilibrium adsorption capacity.

The superior enrichment capability of MBC@SiO_2_–SH was also maintained in aquaculture water, despite the presence of more complex matrix components. This stable performance indicates that the thiol-functionalized surface provides selective coordination sites for Cd^2+^, while the porous structure facilitates mass transfer and improves accessibility of active sites. Therefore, MBC@SiO_2_–SH not only captures Cd^2+^ efficiently but also reduces matrix-derived interference, demonstrating its suitability as a pretreatment material for rapid analytical methods [43].

#### 3.4.2. Selective Extraction and Enrichment of Cd^2+^ in Aquatic Product Matrices

Beyond environmental water samples, aquatic products represent another important complex matrix for Cd^2+^ monitoring because they contain abundant proteins, lipids, and other biological components that may interfere with rapid analytical methods. Therefore, the enrichment capability of MBC@SiO_2_–SH was further evaluated for Cd^2+^ extraction from aquatic products. Crab muscle and hepatopancreas samples (Figure S3) were selected as representative aquatic products and spiked with mixed heavy metal standards. After pretreatment with 5% nitric acid, MBC@SiO_2_–SH was introduced to selectively capture and enrich Cd^2+^ from the extracted solutions. Following magnetic separation and elution, the recovered Cd^2+^ concentration was determined by ICP-MS.

As summarized in Table 5, MBC@SiO_2_–SH achieved Cd^2+^ recovery rates of 88.90–93.29% in crab muscle and 79.94–88.49% in hepatopancreas samples, demonstrating efficient enrichment performance in complex biological matrices. The satisfactory recoveries indicate that MBC@SiO_2_–SH can effectively overcome matrix interference caused by proteins and organic components, enabling reliable Cd^2+^ extraction prior to subsequent detection. In addition to Cd^2+^, MBC@SiO_2_–SH also exhibited adsorption capability toward Pb^2+^, whereas the extraction efficiencies for Zn^2+^, Cu^2+^, and Cr^3+^ were relatively lower. This difference suggests that the abundant thiol groups on MBC@SiO_2_–SH provide preferential coordination toward Cd^2+^ [22],thereby contributing to selective enrichment in complex aquatic product matrices.

Overall, these results demonstrate that MBC@SiO_2_–SH functions not only as an adsorption material but also as an effective enrichment medium for diverse complex matrices. Through selective capture, magnetic separation, matrix cleanup, and analyte concentration, the proposed enrichment strategy provides a critical bridge between sample pretreatment and rapid CLIA detection. This feature distinguishes the present work from conventional adsorption studies and highlights its practical value for trace Cd^2+^ analysis [59].

#### 3.4.3. Column-Based Preconcentration and Enrichment Factor Evaluation

To further translate the enrichment capability into a practical sample pretreatment strategy, MBC@SiO_2_–SH was integrated into a fixed-bed column for continuous Cd^2+^ preconcentration (Figure S4). Compared with batch adsorption, column enrichment enables continuous processing of large-volume samples and provides a direct approach for increasing analyte concentration before detection [60].

Lake water and pond water with an initial Cd^2+^ concentration of 30 mg/L were treated under varying flow rates (1, 2, and 5 mL/min) and adsorbent packing amounts (0.6, 1, and 2 g). As illustrated in Figure 7c,d, the highest removal efficiency was achieved at the lowest flow rate of 1 mL/min, while the efficiency decreased at 5 mL/min. This trend indicates that longer contact time between the adsorbent and contaminants at lower flow rates promotes more complete adsorption and higher removal efficiency. As the adsorption process proceeded, the removal rate gradually declined, and the residual Cd^2+^ concentration in the effluent increased, primarily due to the gradual saturation of available adsorption sites on MBC@SiO_2_–SH. Under optimized conditions (flow rate: 1 mL/min; adsorbent loading: 2 g), the enrichment column achieved adsorption capacities of 49.69 mg/g and 49.13 mg/g for lake water and aquaculture water, respectively. Following enrichment, the captured Cd^2+^ was eluted using 50 mL of 0.1 M EDTA-2NaCa·H_2_O, and the eluates were collected for subsequent CLIA analysis. Based on the initial sample volume (2 L) and final elution volume (50 mL), the enrichment factor (EF) was calculated to be approximately 40.

The enrichment factor of approximately 40 substantially increased the effective Cd^2+^ concentration while simultaneously reducing matrix interference, providing a favorable pretreatment step for subsequent CLIA. Unlike conventional adsorption-based removal approaches, the proposed strategy utilizes MBC@SiO_2_–SH as a selective enrichment medium to convert trace Cd^2+^ from large-volume samples into concentrated eluates suitable for rapid detection.

Based on the intrinsic CLIA detection limit (0.28 μg/L) and the enrichment factor of approximately 40 obtained under the optimized conditions, the effective method detection limit in the original sample was theoretically reduced to approximately 0.007 μg/L after enrichment. Although the intrinsic sensitivity of the immunoassay remained unchanged, selective magnetic preconcentration substantially lowered the detectable concentration in the original matrix by concentrating Cd^2+^ into a smaller elution volume while simultaneously reducing matrix interference. Therefore, the enrichment process effectively extends the practical sensitivity of the rapid CLIA platform toward trace-level analysis. This enrichment-assisted strategy effectively bridges the sensitivity limitation of rapid immunoassays and provides a practical platform for trace Cd^2+^ monitoring in complex environmental matrices [61].

### 3.5. Enrichment-Assisted Rapid CLIA Determination

To evaluated whether this pretreatment strategy could translate into improved analytical performance for rapid Cd^2+^ determination. The enriched Cd^2+^ eluates obtained from environmental water and aquatic product samples were directly subjected to CLIA, and the analytical performance of the proposed enrichment-assisted method was systematically evaluated in terms of repeatability, specificity, accuracy, and practical applicability.

#### 3.5.1. The Repeatability, Stability and Specificity

The repeatability and stability of the enrichment-assisted CLIA method were first evaluated to assess the reliability of the analytical procedure. Calibration curves were obtained over four consecutive days, and the coefficients of variation (CVs) of relative light unit (RLU) responses at different Cd^2+^ concentration levels were below 10% (Table S2), indicating satisfactory repeatability of the proposed method. The storage stability of the CLIA reagents was further investigated by monitoring signal variation during storage at −4 °C. No significant decrease in RLU responses was observed, demonstrating good stability of the detection system and its suitability for routine analysis.

The specificity of the proposed assay was evaluated by assessing its cross-reactivity (CR) with other metal ions, including Li^+^, Na^+^, Mg^2+^, Al^3+^, K^+^, Ca^2+^, Mn^2+^, Fe^3+^, Co^2+^, Ni^2+^, Cu^2+^, Zn^2+^, Rb^+^, Sr^2+^, Ag^+^, Ba^2+^, Hg^2+^, Pb^2+^, and Cd^2+^. Cross-reactivity was calculated according to the following equation: CR (%) = (IC_50_ of Cd^2+^/IC_50_ of the interfering ion) × 100%. As shown in Table S3, all tested ions exhibited negligible cross-reactivity, confirming the high specificity of the proposed assay.

#### 3.5.2. Validation with Spiked Real Samples

To further evaluate the practical applicability of the enrichment-assisted CLIA method, recovery experiments were conducted using environmental water and aquatic product samples with different Cd^2+^ concentrations. For environmental water samples, four Cd^2+^ levels (0.5, 1, 5, and 10 μg/L) were selected for spiking experiments. For aquatic product samples, four concentration levels (50, 100, 200, and 450 μg/kg) were investigated (Table 6). Each concentration level was analyzed in triplicate.

The analytical results obtained by the proposed method were further compared with those obtained by ICP-MS using a paired Student’s *t*-test. No statistically significant differences were observed between the two methods for either environmental water samples (t = 0.949, *p* = 0.41) or aquatic product samples (t = −0.541, *p* = 0.63), indicating good agreement between the proposed homogeneous CLIA and the reference ICP-MS method.

#### 3.5.3. Comparison with Previously Reported Methods

The analytical performance of the proposed strategy was further compared with previously reported Cd^2+^ detection methods (Table 7). Conventional ELISA and immunochromatographic methods generally provide good sensitivity but may suffer from lengthy incubation procedures, insufficient matrix tolerance, or limited enrichment capability. In contrast, the present enrichment-assisted CLIA strategy combines selective magnetic enrichment with rapid homogeneous detection, achieving advantages in preconcentration factor, detection sensitivity, analysis efficiency, and applicability to complex environmental water and aquatic product matrices.

In contrast, the proposed strategy integrates selective magnetic enrichment with homogeneous CLIA, achieving rapid analysis while maintaining high sensitivity and accuracy. The enrichment process effectively increases the concentration of Cd^2+^ in the final eluate and simultaneously removes matrix-derived interference, enabling reliable detection in complex environmental and food samples. These advantages significantly improve the practicality of immunoassay-based Cd determination by reducing both sample preparation time and operational complexity without sacrificing analytical sensitivity. Unlike conventional signal amplification strategies, the improvement in analytical sensitivity originated primarily from increasing the analyte concentration available for immunodetection. Therefore, selective magnetic enrichment extends the practical detection capability of rapid CLIA without altering the intrinsic performance of the immunoassay itself.

## 4. Conclusions

In this study, a thiol-functionalized silica-coated magnetic biochar (MBC@SiO_2_–SH) was successfully developed and integrated with a CLIA for rapid determination of trace Cd^2+^ in environmental water and aquatic products. Benefiting from its abundant thiol coordination sites, large specific surface area, and excellent magnetic responsiveness, MBC@SiO_2_–SH exhibited high adsorption capacity, remarkable selectivity, and good regeneration performance. Mechanistic analyses confirmed that Cd^2+^ adsorption was dominated by Cd–S coordination. Under the optimized conditions, the enrichment column achieved a preconcentration factor of approximately 40, providing efficient matrix cleanup and selective enrichment prior to CLIA. The integrated method achieved limits of detection of 0.007 ng/mL for environmental water and 1.4 ng/g for aquatic products, with recoveries of 92.26–118.64% and 92.41–108%, respectively, and showed no significant difference from ICP-MS measurements. In addition, the enrichment pretreatment itself afforded Cd^2+^ recoveries of 88.90–93.29% in crab muscle and 79.94–88.49% in hepatopancreas samples, demonstrating its applicability to complex biological matrices. Overall, the principal contribution of this work is the integration of selective thiol-based magnetic enrichment, matrix purification, and homogeneous CLIA into a single analytical workflow, which improves the practical trace-detection capability of CLIA without relying on detector-side signal amplification. This strategy provides a simple, rapid, and sensitive approach for Cd^2+^ monitoring in complex environmental and aquatic-product matrices and highlights selective sample pretreatment as a key component of practical rapid analysis.

## Figures and Tables

**Table 1:** Textural parameters of biochar materials.

Materials	BET Surface Area (m^2^·g^−1^) ^a^	Langmuir Surface Area (m^2^·g^−1^)	Average Pore Diameter (nm)	Pore Volume (cm^3^·g^−1^)	Mesopore Area (m^2^·g^−1^)	Mesopore Diameter (nm) ^a^	Mesopore Volume (cm^3^·g^−1^) ^a^	Micropore Area (m^2^·g^−1^)	Micropore Volume (cm^3^·g^−1^) ^a^
BC	35.652	94.735	4.898	0.044	13.217	10.235	0.034	22.775	0.010
MBC@SiO_2_-SH	102.413	756.211	8.710	0.223	94.604	9.230	0.218	10.114	0.003

^a^ The specific surface area, the mesopore volume, and the micropore volume were determined by the methods of the Brunauer–Emmett–Teller (BET) method, the Barrett–Joyner–Halenda (BJH) method, and the t-plot method, respectively.

**Table 2:** Kinetic model parameters for the adsorption of Cd^2+^ onto BC and MBC@SiO_2_-SH in water.

Materials	*q_e,exp_* (mg·g^−1^)	Pseudo-First-Order			Pseudo-Second-Order			Avrami-Fractional-Order				Elovich		
		*q_e,cal_* (mg·g^−1^)	*k*_1_ (min^−1^)	*R* ^2^	*q_e,cal_* (mg·g^−1^)	*k*_2_ (g·mg^−1^·min^−1^)	*R* ^2^	*q_e,cal_* (mg·g^−1^)	*k*_3_	*n*	*R* ^2^	*α* (mg·g^−1^·min^−1^)	*β* (mg·g^−1^)	*R* ^2^
BC	17.26	15.22	1.472	0.975	15.26	0.132	0.869	14.62	15.217	8.385	0.956	7005.799	0.894	0.999
MBC@SiO_2_-SH	46.65	46.53	0.007	0.999	49.01	2.715 × 10^−4^	0.898	47.32	0.007	0.772	0.999	6.063	0.142	0.998

Note: *q_e,exp_*—*q_e,experimental_*; *q_e,cal_*—*q_e,calculated_*.

**Table 3:** Isotherm parameters for Cd^2+^ adsorption onto BC and MBC@SiO_2_-SH at 298.15 K.

Isotherm Models	Parameters	BC	MBC@SiO_2_-SH
Langmuir	*q_max_* (mg/g)	21.460	46.407
	*K_L_* (L/mg)	0.776	0.258
	*R* ^2^	0.768	0.997
Freundlich	*K_F_* (mg/g) (L/mg)^1/n^	4.266	13.734
	*n_F_*	0.434	0.442
	*R* ^2^	0.494	0.584
Temkin	*K_T_* (L/mg)	14.971	136.055
	*b_T_* (kJ/mol)	659.140	438.729
	*R* ^2^	0.865	0.997
D-R	*q_max_* (mg/g)	16.657	33.375
	*K_D-R_* (L/mg)	0.068	0.023
	*β* (mol^2^/kJ^2^)	1.11 × 10^−2^	3.75 × 10^−3^
	*E* (kJ/mol)	6.72	11.55
	*R* ^2^	0.847	0.940

**Table 4:** Thermodynamics parameters for Cd^2+^ uptake by BC and MBC@SiO_2_-SH.

Materials	T (°C)	T (K)	ln K°	∆G^0^ (KJ/mol)	∆H^0^ (KJ/mol)	∆S^0^ (J·mol^−1^·K^−1^)
BC	15	288.15	6.93	−16.59	11.96	99.19
	25	298.15	7.14	−17.70		
	35	308.15	7.22	−18.50		
	45	318.15	7.42	−19.64		
MBC@SiO_2_-SH	15	288.15	8.92	−21.37	27.91	171.60
	25	298.15	9.44	−23.41		
	35	308.15	9.86	−25.26		
	45	318.15	10.00	−26.44		

Note: K° represents the dimensionless standard thermodynamic equilibrium constant calculated by $K^{0}=(q_{e}/q^{0})/(C_{e}/C^{0})$, where $q^{0}$ = 1 mol kg^−1^ and $C^{0}$ = 1 mol L^−1^.

**Table 5:** Recovery rate of Cd^2+^ from Chinese mitten crab (*Eriocheir sinensis*) muscle and crab roe/paste using MBC@SiO_2_-SH pretreatment.

Metal Ion	Muscle (Wet Weight)						
	Concentration (μg/kg)	Spiked Concentration (μg/kg)	Recovery Rate (%)	Spiked Concentration (μg/kg)	Recovery Rate (%)	Spiked Concentration (μg/kg)	Recovery Rate (%)
Cd^2+^	46.09	0.5 × 10^3^	84.90	2 × 10^3^	88.67	10 × 10^3^	93.29
Pb^2+^	6.67		86.04		83.20		82.48
Cu^2+^	27.05		35.16		35.25		35.16
Zn^2+^	63.35		15.67		17.17		17.83
Cr^3+^	13.40		15.54		15.33		15.31
Metal ion	Crab roe/paste (Wet weight)						
	Concentration (μg/kg)	Spiked concentration (μg/kg)	Recovery rate (%)	Spiked concentration (μg/kg)	Recovery rate (%)	Spiked concentration (μg/kg)	Recovery rate (%)
Cd^2+^	53.35	0.5 × 10^3^	79.94	2 × 10^3^	83.24	10 × 10^3^	88.49
Pb^2+^	11.14		79.82		76.17		75.42
Cu^2+^	44.73		26.54		26.68		27.06
Zn^2+^	72.27		9.75		10.59		10.96
Cr^3+^	19.77		4.79		4.85		4.99

**Table 6:** Spiked Recovery Analysis of Real Samples.

Matrix	Spiked Concentration (μg/kg)	ICP-MS (μg/kg)	CLIA (μg/kg)	Recovery	*t*-Test, (0.05,3)	*p*
water samples (*n* = 3)	0.5	0.51 ± 0.03	0.52 ± 0.06	94.57–118.64%	0.949	0.41
	1	0.99 ± 0.02	1 ± 0.07	92.26–105.74%		
	5	5.01 ± 0.03	4.98 ± 0.02	99.31–100.03%		
	10	9.99 ± 0.18	10.29 ± 0.27	100.15–105.62%		
Aquatic Products (*n* = 3)	50	50.51 ± 0.45	48.43 ± 1.58	93.70–100.02%	−0.541	0.63
	100	101.33 ± 0.97	99.78 ± 5.62	94.16–105.40%		
	200	203.18 ± 1.78	205.34 ± 10.66	97.34–108%		
	450	447.76 ± 4.12	447.19 ± 31.36	92.41–106.34%		

**Table 7:** Summary of similar detection methods for Cd.

Method	Sample Matrix	Pretreatment Strategy	Limit of Detection	Test Time	Reference
ELISA	Wheat, rice, apple juice, tea	Acid digestion, overnight boiling	0.2 ng/mL	18 h	[62]
ELISA	Water	Direct analysis	0.05 ng/mL	6.5 h	[63]
ICP-MS	Water	Direct analysis	0.375 ng/mL	--	[63]
ICP-MS	Brown rice	Microwave digestion, isolation column	3 ng/mL	--	[64]
ICP-MS	Wheat, eggplant	Ion-exchange purification	2.5 ng/mL	40 min	[65]
CLIA	Water, Aquatic Products	5% HNO_3_, 5 min	0.007 ng/mL, 1.4 ng/g	30 min	This work

## Data Availability

The raw data supporting the conclusions of this article will be made available by the authors on request.

## Supplementary Materials

The following supporting information can be downloaded at: https://www.mdpi.com/article/10.3390/toxics14090785/s1, Text S1. Characterization of biochar; Text S2. Cd^2+^ analysis in water and aquatic products; Text S3. Adsorption models of Cd^2+^; Figure S1. The adsorption thermodynamics of BC and MBC@SiO_2_-SH in aqueous solution; Figure S2. Water samples from lake and aquaculture pond; Figure S3. Sampling of Chinese mitten crabs; Figure S4. Column-type experimental apparatus. Table S1. Comparison of Cd(II) adsorption performance of carbon-based adsorbents; Table S2. The repeatability and stability test; Table S3. Specificity evaluation of this method. References [66,67,68,69,70,71] are cited in Supplementary Materials.
