# Supplementary material for: Thiol-Functionalized Magnetic Biochar-Mediated Matrix Cleanup and Selective Enrichment for Trace-Level Rapid Determination of Cadmium in Environmental Water and Aquatic Products
Source: Toxics. 2026 Sep 5;14(9):785. doi: 10.3390/toxics14090785 (PMC13611515; doi:10.3390/toxics14090785)
Supplement: Supplementary file 1 [file toxics-14-00785-s001.zip › toxics-4512955-supplementary.pdf]

Supplementary Information

Thiol-functionalized Magnetic Biochar-Mediated Matrix Cleanup and Selective Enrichment for Trace-Level Rapid Determination of Cadmium in Environmental Water and Aquatic Products

Content

Texts:

Text S1. Characterization of biochar

Text S2. Cd^{2+} analysis in water and aquatic products

2.1 Sample pretreatment

2.1.1 Water sample

2.1.2 Aquatic product sample

2.2 Instrumental analysis

2.3 Quality assurance and quality control

Text S3. Adsorption models of Cd^{2+}

3.1 Adsorption kinetics

3.2 Adsorption isotherms

3.3 Adsorption thermodynamics

Figures:

Figure S1. The adsorption thermodynamics of BC and MBC@SiO_2-SH in aqueous solution.

Figure S2. Water samples from lake and aquaculture pond.

Figure S3. Sampling of Chinese mitten crabs.

Figure S4. Column-type experimental apparatus.

Tables:

Table S1. Comparison of Cd(II) adsorption performance of carbon-based adsorbents

Table S2. The repeatability and stability test.

Table S3. Specificity evaluation of this method.

Text S1. Characterization of biochar

The thermal stability of the materials was analyzed using a thermogravimetric analyzer (STA 449 F5, Netzsch, Germany). The surface microstructure of sample was analyzed using the scanning electron microscope (GeminiSEM 300, ZEISS, Germany). The Brunauer–Emmett–Teller (BET) surface area, pore size, and pore volume of the sample were analyzed with an Automatic Surface Area and Pore Analyzer (ASAP 2460, Micromeritics, USA). Vibrating Sample Magnetometer (VSM) primarily measures the magnetic properties of biochar to determine their magnetic classification (LakeShore 8604, USA). The biochar structure was characterized by X-ray Diffraction (Ultima IV, Rigaku, Japan) with Cu Kα radiation, scanning was run from 5° to 90° (2θ). The surface functional group of the sample was analyzed with an FT-IR spectrophotometer (Nicolet iS20, Thermo Fisher Scientific, USA). The surface chemical states of the catalyst were analyzed by X-ray photoelectron spectrometry (XPS) using a K-Alpha spectrometer with Al Kα radiation (Nexsa, Thermo Fisher Scientific, USA).

Text S2. Cd^{2+} analysis in water and aquatic products

2.1 Sample pretreatment

2.1.1 Water sample

We employed vacuum filtration and membrane filtration in conjunction with Inductively Coupled Plasma Mass Spectrometry (ICP-MS) to detect heavy metals in the water samples. Specifically, after collection, immediately filter the soluble element sample using a 0.45 μm filter membrane. Discard the initial 50 mL of filtrate. Rinse the sample bottle with a small amount of filtrate, then collect the required volume of filtrate into the sample bottle. Add an appropriate amount of nitric acid to adjust the acidity to pH < 2. Subsequently, the extraction syringe was injected into the ICP-MS inlet for further analysis.

2.1.2 Aquatic product sample

Original Method: aquatic tissue samples (0.5±0.01 g) were added to 5 mL HNO_3, preheated at 110℃ for 30 min, then microwave digested at 190℃, 1000 W for 25 min (ramp) + 25 min (hold). Cool to room temperature, evaporate acid to approximately 1 mL on a hot plate, cool to room temperature, dilute to 50 mL, filter through a 0.22 μm membrane, and analyze by instrument.

New Method: a 5 mL aliquot of the aquatic product sample (0.5 g), pretreated with 5% nitric acid, was transferred into a glass vial. The pH was adjusted to approximately 8.0 by adding 0.6 M NaOH, and the final volume was brought to 10 mL. Then, 40 mg of adsorbent was introduced, and the mixture was magnetically stirred for 35 min. The adsorbent was then collected and immersed in 0.1 M HCl for 10 min, followed by washing with 0.1 M EDTA-2NaCa·H_2O and deionized water. The eluates were combined to a total volume of 20 mL, and the Cd^{2+} concentration was determined by ICP-MS.

2.2 Instrumental analysis

Before starting the instrument, perform collision reaction cell maintenance and sample introduction maintenance and optimization. Atomization chamber temperature: 2℃; Sampling depth: 7.5 mm; RF power: 1550 W; Plasma gas (argon) flow rate: 15.00 L/min; auxiliary gas flow rate: 1.00 L/min; atomization gas flow rate: 1.00 L/min; compensation/dilution gas flow rate: 1.00 L/min.

2.3 Quality assurance and quality control

The limits of detection (LODs) and limits of quantification (LOQs) were defined as the

concentrations corresponding to a signal-to-noise ratio of 3 and 10. The LODs and LOQs of Cd^{2+} in water were 0.05 μg/L and 0.2 μg/L, respectively.

Recoveries of Cd^{2+} were 80-120% in the water samples. Precision was assessed using the relative standard deviation (RSD) of six replicate treatments. The methodology was generally well reproducible, with RSDs < 20%.

Text S3. Adsorption models of Cd^{2+}

3.1 Adsorption kinetics

The removal rate (R_r) and adsorption capacity were calculated as follows:

$$R_r = \frac{(C_0\text{-}C_e)}{C_0} \times 100\% \tag{1}$$

Adsorption capacity at moment t:

$$q_t = \frac{(C_0\text{-}C_t)}{m} \times V \tag{2}$$

Where q_e (μg/g) and q_t (μg/g)) are the number of pollutants adsorbed by the biochars at equilibrium and at time t, respectively; C_0 (μg/L) and C_e (μg/L) represent the initial and equilibrium concentrations of pollutants, respectively.

The equations and associated parameters for the three dynamics are given below:

Pseudo-first-order:

$$q_t = q_e(1 - e^{-k_1 t}) \tag{3}$$

Pseudo-second-order:

$$q_t = \frac{t k_2 q_e^2}{1 + t q_e k_2} \tag{4}$$

Avrami fractional-order:

$$q_t = q_e\left[1 - e^{-(k_3 t)^n}\right] \tag{5}$$

Elovich:

$$q_t = \frac{1}{\beta}\ln(\alpha\beta t + 1) \tag{6}$$

Intra-particle diffusion:

$$q_t = k_i t^{0.5} + C_i \tag{7}$$

The variables in question are as follows: t is the adsorption time, min; q_e denotes the equilibrium adsorption amount, μg/g; q_t denotes the adsorption amount at time t, μg/g; k_1 is the proposed first-order kinetic coefficient, min^{-1}; k_2 denotes the proposed second order adsorption rate constant, g/μg·min $^{-1}$; k_3 denotes the constant, and n is the Avrami index, generally in the range 1 to 4; α (mol/g·min) and β as the elovich constants represent the initial adsorption rate and the desorption coefficient; k_i and C_i are the pore rate constant (g/μg·$min^{-0.5}$) and the constant, respectively.

3.2 Adsorption isotherms

To further investigate the adsorption pattern and adsorption mechanism of antibiotics on different biochars, adsorption isothermal equations were fitted with the Langmuir and Freundlich models, respectively.

Langmuir:

$$q_e = \frac{K_L q_{max} C_e}{1 + K_L C_e} \tag{8}$$

Freundlich:

$$q_e = K_F C_e^{\frac{1}{n_F}} \tag{9}$$

Sips:

$$q_e = \frac{K_S C_e^{n_S}}{1 + a_S C_e^{n_S}} \tag{10}$$

Temkin:

$$q_e = \frac{\mathrm{RT}}{b_T} \ln(K_T C_e) \tag{11}$$

where, the equilibrium and saturation quantity of geosmin captured by biochars are represented by q_e and q_{max}, K_L, K_F, and K_S are correlated with the coefficients of models, n_F and n_S mean corresponding indexes, K_T is the equilibrium binding constant, b_T is Temkin constant related to heat of adsorption, R is the gas constant (8.314 J/mol·K), C_e are the concentration of the un-adsorbed geosmin at equilibrium, while T is the absolute temperature.

3.3 Adsorption thermodynamics

The adsorption thermodynamic parameters were investigated at temperatures of 288.15 K, 298.15 K, 308.15 K, and 318.15 K. The changes in Gibbs free energy (ΔG^0), enthalpy (ΔH^0), and entropy (ΔS^0), which are related to the feasibility and nature of the adsorption process, are expressed using the following equations.

$$\Delta G^0 = -\mathrm{RT}\ln(K_0) = \Delta H^0 - T\Delta S^0 \tag{12}$$

$$\ln K_0 = \frac{\Delta S^0}{R} - \frac{\Delta H^0}{RT} \tag{13}$$

$$K_0 = \frac{q_e}{C_e} \tag{14}$$

in which, K_0 as a thermodynamic equilibrium constant can be calculated by Langmuir isotherm; R is ideal gas constant, 8.314 J/(mol·K); q_e is the equilibrium adsorption amount (μg/g), and C_e is the equilibrium concentration (μg/L); T is thermodynamic temperature (K).

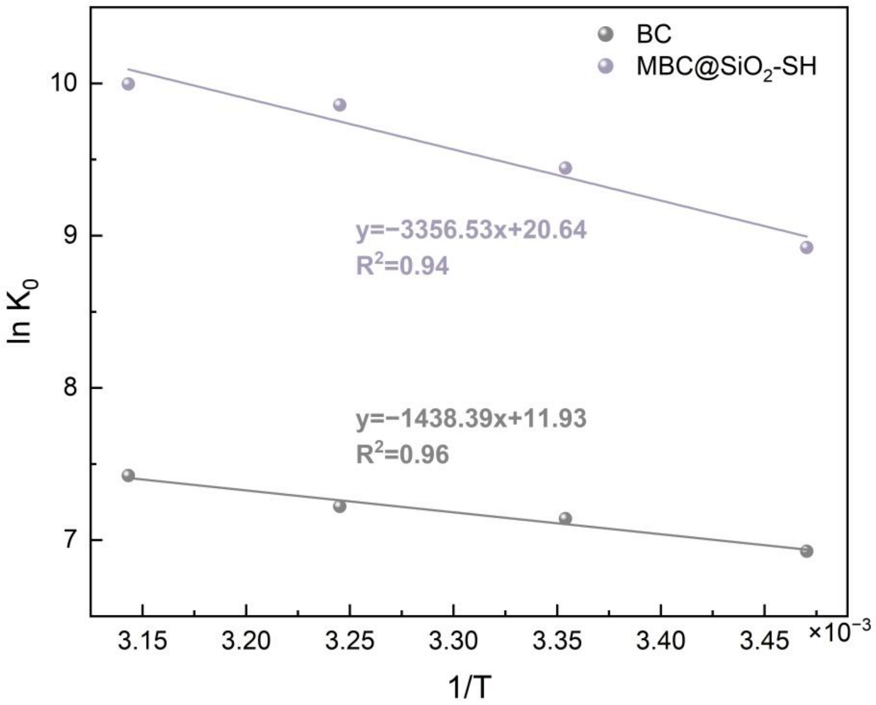

Figure S1. The adsorption thermodynamics of BC and MBC@SiO_2-SH in aqueous solution.

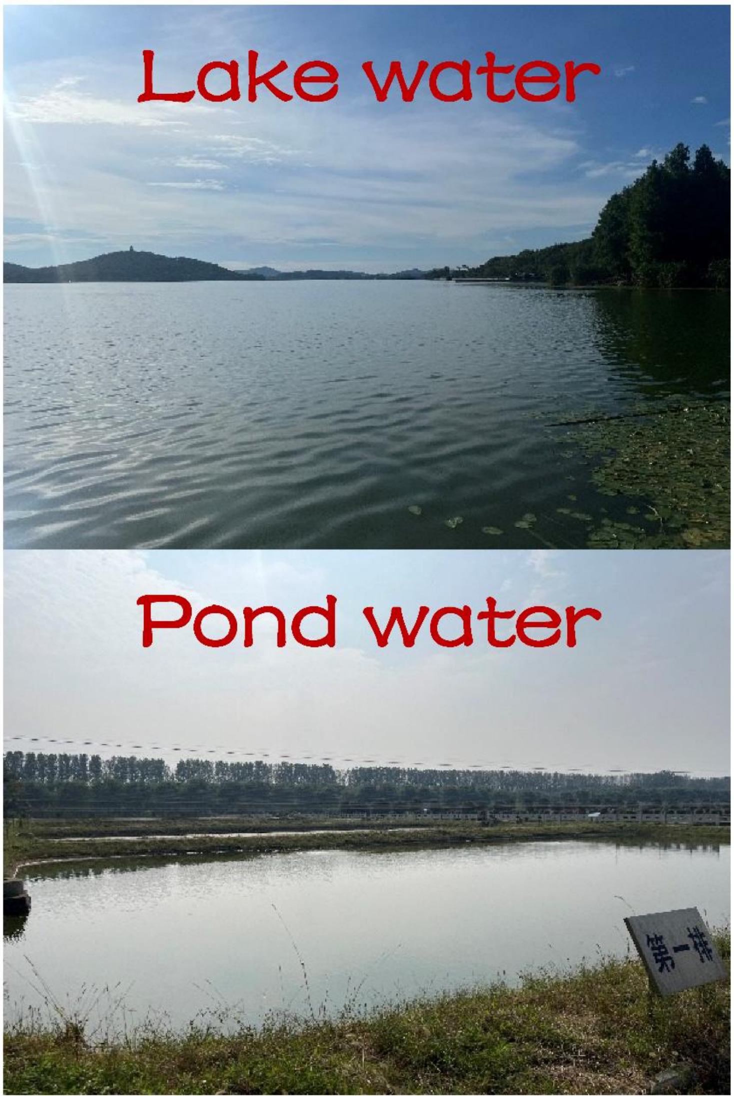

Figure S2. Water samples from lake (Li Lake, located in Wuxi, Jiangsu Province) and aquaculture pond (fish farming pond, in Wuxi, Jiangsu Province).

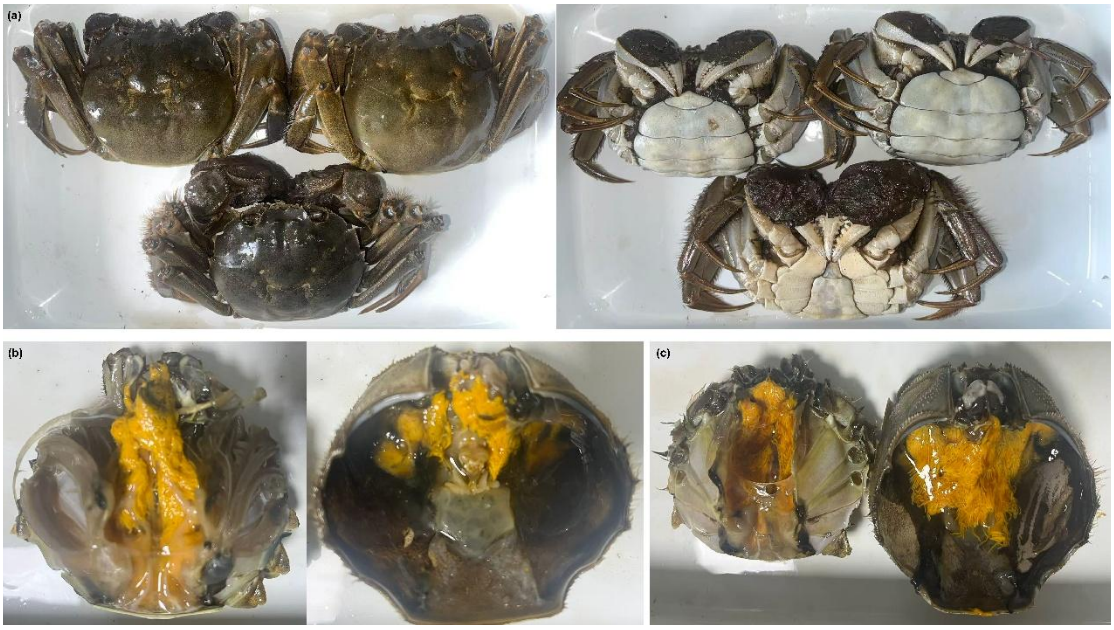

Figure S3. Sampling of (a) Chinese mitten crabs (*Eriocheir sinensis*). (b) Crab roe from female crabs and (b) crab paste from male crabs.

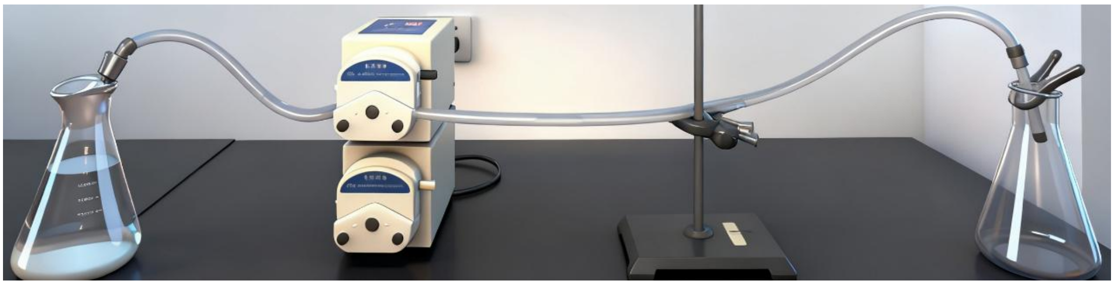

Figure S4. Column-type experimental apparatus.

Table S1. Comparison of Cd(II) adsorption performance of carbon-based adsorbents

Adsorbent	Initial Cd(II) (mg/L)	Dosage (g/L)	pH	Contact time	Maximum adsorption capacity / removal efficiency	Reference
Cotton-straw biochar	/	/	2–8	Equilibrium within 40 min	10.14 mg/g	Guo et al. (2013)
Activated biochar from torrefied loblolly pine	/	/	7.5 ± 0.1	/	167.3 mg/g	Park et al. (2017)
Magnetic corn-straw biochar (MBC800-0.6_{300})	/	/	Variable	/	46.90 mg/g	Khan et al. (2020)
Tea-branch biochar (TBB700)	10–300	2	6	5 min–24 h	86.55 mg/g	Han et al. (2021)
Blue-algae-derived biochar (BC600-2)	/	/	/	/	135.7 mg/g	Liu et al. (2021)
Sewage-sludge/$CaSO_4$ co-pyrolysis biochar (CSSB)	/	0.4–6*	/	0–24 h	145.4 mg/g	Liu et al. (2021)
MBC@SiO_2-SH (This study)	30	0.6	5	12 h	50.22 mg/g	This study
MBC@SiO_2-SH (This study)	50	0.6	5	12 h	82.96 mg/g	This study

Table S2. The repeatability and stability test.

μg/L	Day1	Day2	Day3	Day4	Mean	CV%
0	491819	441380	449729	453485	459103	4.88
1.37	410805	394430	349519	398650	388351	6.90
4.12	326652	317961	308328	315909	317213	2.38
12.35	201952	195291	191426	188781	194362	2.94
37.04	93895	90468	93385	89636	91846	2.30
111.11	39844	39548	39311	37903	39152	2.20
333.33	21437	20247	19026	20195	20226	4.87
1000	15104	17014	17141	16431	16422	5.68

Table S3. Specificity evaluation of this method

Metal	IC_{50} (ng mL^{-1})	CR (%)	Metal	IC_{50} (ng mL^{-1})	CR (%)
Cd^{2+}	0.20	100.00	Rb^{+}	>1000	<0.02
Li^{+}	>1000	<0.02	Sr^{2+}	>1000	<0.02
Na^{+}	>1000	<0.02	Ag^{+}	>1000	<0.02
Mg^{2+}	>1000	<0.02	Ba^{2+}	>1000	<0.02
Al^{3+}	>1000	<0.02	Hg^{2+}	>1000	<0.02
K^{+}	>1000	<0.02	Pb^{2+}	>1000	<0.02
Ca^{2+}	>1000	<0.02	Co^{2+}	>1000	<0.02
Mn^{2+}	>1000	<0.02	Cu^{2+}	>1000	<0.02
Fe^{3+}	>1000	<0.02	Zn^{2+}	>1000	<0.02
Ni^{2+}	>1000	<0.02			

References

1. Guo, W.-J., Liang, X.-F., Lin, D.-S., Xu, Y.-M., Wang, L., Sun, Y.-B., Qin, X. 2013. Adsorption of Cd2+ on biochar from aqueous solution. Huan Jing Ke Xue 34(9), 3716–3721. PMID: 24289029.
2. Park, C.M., Han, J., Chu, K.H., Al-Hamadani, Y.A.J., Her, N., Heo, J., Yoon, Y. 2017. Influence of solution pH, ionic strength, and humic acid on cadmium adsorption onto activated biochar: Experiment and modeling. Journal of Industrial and Engineering Chemistry 48, 186–193. https://doi.org/10.1016/j.jiec.2016.12.038.
3. Khan, Z.H., Gao, M., Qiu, W., Islam, M.S., Song, Z. 2020. Mechanisms for cadmium adsorption by magnetic biochar composites in an aqueous solution. Chemosphere 246, 125701. https://doi.org/10.1016/j.chemosphere.2019.125701.
4. Han, C., Wang, M., Ren, Y., Zhang, L., Ji, Y., Zhu, W., Song, Y., He, J. 2021. Characterization of pruned tea branch biochar and the mechanisms underlying its adsorption for cadmium in aqueous solution. RSC Advances 11, 26832–26843. https://doi.org/10.1039/D1RA04235A.
5. Liu, P., Rao, D., Zou, L., Teng, Y., Yu, H. 2021. Capacity and potential mechanisms of Cd(II) adsorption from aqueous solution by blue algae-derived biochars. Science of the Total Environment 767, 145447. https://doi.org/10.1016/j.scitotenv.2021.145447.
6. Liu, L., Yue, T., Liu, R., Lin, H., Wang, D., Li, B. 2021. Efficient absorptive removal of Cd(II) in aqueous solution by biochar derived from sewage sludge and calcium sulfate. Bioresource Technology 336, 125333. https://doi.org/10.1016/j.biortech.2021.125333.
